# Deep RNA Sequencing of the Skeletal Muscle Transcriptome in Swimming Fish

**DOI:** 10.1371/journal.pone.0053171

**Published:** 2013-01-08

**Authors:** Arjan P. Palstra, Sergi Beltran, Erik Burgerhout, Sebastiaan A. Brittijn, Leonardo J. Magnoni, Christiaan V. Henkel, Hans J. Jansen, Guido E. E. J. M. van den Thillart, Herman P. Spaink, Josep V. Planas

**Affiliations:** 1 Departament de Fisiologia i Immunologia, Facultat de Biologia, Universitat de Barcelona and Institut de Biomedicina de la Universitat de Barcelona (IBUB), Barcelona, Spain; 2 Unitat de Bioinformàtica, Centres Cientifics i Tecnològics (CCiT-UB), Universitat de Barcelona, Barcelona, Spain; 3 Molecular Cell Biology, Institute of Biology, Leiden University (IBL), Sylvius Laboratory, Leiden, The Netherlands; 4 ZF-screens, Leiden, The Netherlands; University of North Carolina at Chapel Hill, United States of America

## Abstract

Deep RNA sequencing (RNA-seq) was performed to provide an in-depth view of the transcriptome of red and white skeletal muscle of exercised and non-exercised rainbow trout (*Oncorhynchus mykiss)* with the specific objective to identify expressed genes and quantify the transcriptomic effects of swimming-induced exercise. Pubertal autumn-spawning seawater-raised female rainbow trout were rested (n = 10) or swum (n = 10) for 1176 km at 0.75 body-lengths per second in a 6,000-L swim-flume under reproductive conditions for 40 days. Red and white muscle RNA of exercised and non-exercised fish (4 lanes) was sequenced and resulted in 15–17 million reads per lane that, after *de novo* assembly, yielded 149,159 red and 118,572 white muscle contigs. Most contigs were annotated using an iterative homology search strategy against salmonid ESTs, the zebrafish *Danio rerio* genome and general Metazoan genes. When selecting for large contigs (>500 nucleotides), a number of novel rainbow trout gene sequences were identified in this study: 1,085 and 1,228 novel gene sequences for red and white muscle, respectively, which included a number of important molecules for skeletal muscle function. Transcriptomic analysis revealed that sustained swimming increased transcriptional activity in skeletal muscle and specifically an up-regulation of genes involved in muscle growth and developmental processes in white muscle. The unique collection of transcripts will contribute to our understanding of red and white muscle physiology, specifically during the long-term reproductive migration of salmonids.

## Introduction

Skeletal muscle is an important tissue for whole body metabolic homeostasis and for locomotion. In fish, skeletal muscle may represent approximately half of their body mass and provides the engine for swimming, an intrinsic and characteristic behaviour of this group of vertebrates. From a functional point of view, two types of skeletal muscle can be identified in fish: white skeletal muscle, which is anaerobic and fuels burst swimming, and red skeletal muscle, which is aerobic and fuels sustained swimming [1]. For many fish species, their life history is intimately linked to their ability to perform under swimming-induced exercise conditions that, in turn, is dependent on the functionality of skeletal muscle.

Among migrant fish species, the most extreme exercise conditions are experienced during the anorexic reproductive migration, as performed by salmonid species [2], [3]. Fish that migrate long distances to reach their spawning grounds like salmonids face two major challenges before they can successfully reproduce: to swim and to sexually mature. Recently, we applied exercise experimentally to investigate its effects on sexual maturation in female rainbow trout [4]. The main conclusion of that study was that swimming suppresses ovarian development at the start of vitellogenesis. Swimming requires streamlining of the body and muscle building for optimal performance. However, the progression of oocyte growth (e.g. vitellogenesis) may cause a change in body shape that, in turn, could increase drag resistance, and may also lead to muscle atrophy [5], [6], leading to decreased swimming efficiency. Therefore, long distance migrants need to up-regulate the energetic processes in the muscle that provide fuel for contraction and for muscle growth, and to suppress vitellogenesis: the migration phenotype. When there is a need to start vitellogenesis, the situation in the muscle and the ovary is reversed: the sexual maturation phenotype.

Despite the important role of skeletal muscle for swimming in fish, relatively little is known regarding the molecular events that take place in red and white skeletal muscle in response to swimming-induced activity. In this study, we have used deep RNA sequencing (RNA-seq), a high-throughput transcriptomic approach, to provide an in-depth view of the transcriptome of red and white skeletal muscle in rainbow trout. To the best of our knowledge, our study represents the first application of RNA-seq to the study of the skeletal muscle transcriptome in rainbow trout. We have chosen to use rainbow trout because it is an economically important species for aquaculture and because it displays (facultative) migrant behaviour. Still, in absence of a reference genome of rainbow trout, the nucleotide transcripts (‘reads’) need to be *de novo* assembled to larger groups of sequences representing overlapping regions from the transcriptome (‘contigs’). Contigs can then be annotated using iterative sequence homology searches against known related sequences such as salmonid ESTs or the zebrafish genome. In the present study, we used RNA-seq to catalogue the red and white muscle transcriptome in rainbow trout. We also aimed to investigate the effects of exercise in red and white muscle and, specifically, to focus on muscle building versus muscle wasting processes and on the potential interaction between skeletal muscle and the reproductive axis. The gained information should allow us to determine whether the rainbow trout muscle matched the migration phenotype or the sexual maturation phenotype after the long-term exercise of simulated reproductive migration.

## Materials and Methods

### The Experimental Set-up

The swimming experiment was performed in a 6,000 L swim-flume recently described in Palstra et al. [4]. In short, an oval shaped swim-flume (6.0×4.0×0.8 m) had been placed in a 100 m^2^ climatized room. In one of the straight ends of the swim-flume, a compartment of 2.0×0.7 m was created with two mesh fences (5 cm mesh size). This compartment was divided by a PVC fence that started in the curve. The resulting inner compartment, where the current was null, was used to house the resting group. The outer compartment where the current was maximal was used to house the swimming group. Thus, both groups were subjected to identical conditions except for swimming exercise. Initially, water in the swim-flume was brackish at 10 ‰, created by mixing natural seawater from the Oosterschelde at the Burgersluis as delivered by truck (Van Vugt, Zuilinchem, the Netherlands), with tap water. Flow was created and speed profiles were measured as described before [4] as well as water temperature and water quality parameters that were monitored and controlled.

### Ethics

The swimming experiments as described have been approved by the animal welfare committee (DEC) of Leiden University under number 08107.

### Experimental Fish and Conditions

In order to simulate the natural reproductive conditions of anadromous salmonids, experiments were performed with sea water-raised rainbow trout. Resters and swimmers were unfed during the experiment, seawater was replaced by fresh water and photoperiod was changed as described below. Because both resters and swimmers were experiencing these conditions at the same time in the same set-up, any additional effects in swimming fish would be expected to be caused by swimming only.

Pubertal autumn spawning female rainbow trout (n = 20; ∼50 cm, ∼1.8 kg) were purchased from a Danish exporter (Frederiksvaerk Aleexport, Frederiksvaerk, Denmark) where they had been raised for 2 years in freshwater followed by 4 months in sea water cages at 10 ‰. They were transferred by truck within 16 h directly to the swim-flume at Leiden University (The Netherlands). Fish were randomly divided into a ‘rest’-group (n = 10) and a ‘swim’-group (n = 10). During the following 4 days, the brackish 10 ‰ water was stepwise replaced by freshwater at 16°C and photoperiod was changed from 16L:8D to 8L:16D. Fish were then acclimatized for two days to their new conditions. As in our previous study [4], swimmers were first swum at a speed of 0.33 body-lengths per second (BL/s) that was increased the next day to the final, near optimal speed of 0.75 BL/s (0.34 m/s or 29.4 km/day) and all fish were sampled after 40 days. Fish did not show any signs of fatigue, stress or health problems during 40 days of resting or swimming without feeding. After 40 days, a distance of 1,176 km had been swum continuously in a sustained manner. At sampling, fish were anesthetized using oil of cloves (diluted 1∶10 in ethanol and used at a dosage of 1.5 ml/l), euthanized by decapitation and dissected for red and white muscle tissue from standardized locations. Samples were flash frozen in liquid nitrogen and stored at −80°C for RNA sequencing (RNA-seq) and Q-PCR. In addition, the gonadosomatic index (GSI) was measured from trout at the beginning of the experiment (day 0) and at the termination of the experiment (day 40). Our results show that the GSI of resters at the termination of the 40-day experimental period (GSI = 5.47±0.25%) was significantly higher than that of fish at the beginning of the experimental period (day 0; GSI = 0.58±0.07%), indicating that ovarian development was stimulated by the reproductive conditions. However, no significant differences in FSI were observed between resters and swimmers at the termination of the 40-day experimental period (data not shown).

### RNA Isolation, Library Preparation and Sequencing

Equal parts from each of the ten individual tissue samples per group (red muscle from swimmers, white muscle from swimmers, red muscle from resters, white muscle from resters) were pooled in QIAzol Lysis Reagent (Qiagen Benelux BV, Venlo, The Netherlands). A Qiagen TissueRuptor (Qiagen Benelux BV, Venlo, The Netherlands) was used to cut up the tissue samples and RNA was extracted from each of these pools using the Qiagen miRNeasy Mini Kit (Qiagen Benelux BV, Venlo, The Netherlands) according to the manufacturer’s description. RNA was eluted in 50 µl and quantified by Nanodrop (Thermo Fisher Scientific, Amsterdam, The Netherlands). For each sample a RNA-seq library was prepared with an Illumina mRNA-Seq Sample Preparation Kit according to the manufacturer’s description (Illumina HQ, San Diego CA, USA) and cluster generation was performed. For each library, a single read of 51 nucleotides was performed with each sample group on one lane of a flowcell. The flowcell was run on an Illumina GAIIx sequencer. Image analysis and base calling were done by the Illumina pipeline (Illumina HQ, San Diego CA, USA). The Illumina GA IIx uses the clonally amplified template method resulting in a population of identical templates coupled with the four-colour cyclic reversible termination (CRT) method to compromise nucleotide incorporation, fluorescence imaging and cleavage [7]. Raw RNA-seq data (reads) have been submitted to the NCBI Short Read Archive (http://trace.ncbi.nlm.nih.gov: number SRA051669.1).

### *De novo* Assembly of Contigs and Transcript Quantification

*De novo* assembly of contigs ≥100 nt was performed per tissue (pooled for resters and swimmers for either red or white muscle). The CLC bio Genomics Workbench (version 3.6.5, CLC bio, Aarhus, Denmark) was used to remove low quality reads and nucleotides, assemble mRNA contigs *de novo* and quantify expression. Reads were aligned to the contigs (allowing 1 mismatch and up to 10 alignments per read) and summarized as Reads Per Kilobase (exon model) per Million mapped reads (RPKM) normalized expression values (aligned read count normalized for contig length and total number of aligned reads; [8]). For differential expression between swimmers and resters of each contig, the RPKM value of the swimmers was divided by that of the resters. The result was considered as fold change (fc) in expression between swimmers and resters. Contigs with a fc ≤0.5 were considered to be down-regulated in swimmers, while those with a fc ≥2 were considered to be up-regulated in swimmers. Judging differential expression on basis of such stringent fold change criteria certainly selects biologically meaningful differences, moreover, using fold change as criterium when dealing with high numbers of genes like with RNA-seq results in lower false discovery rates than when using statistical t-tests [9].

**Figure 1:**
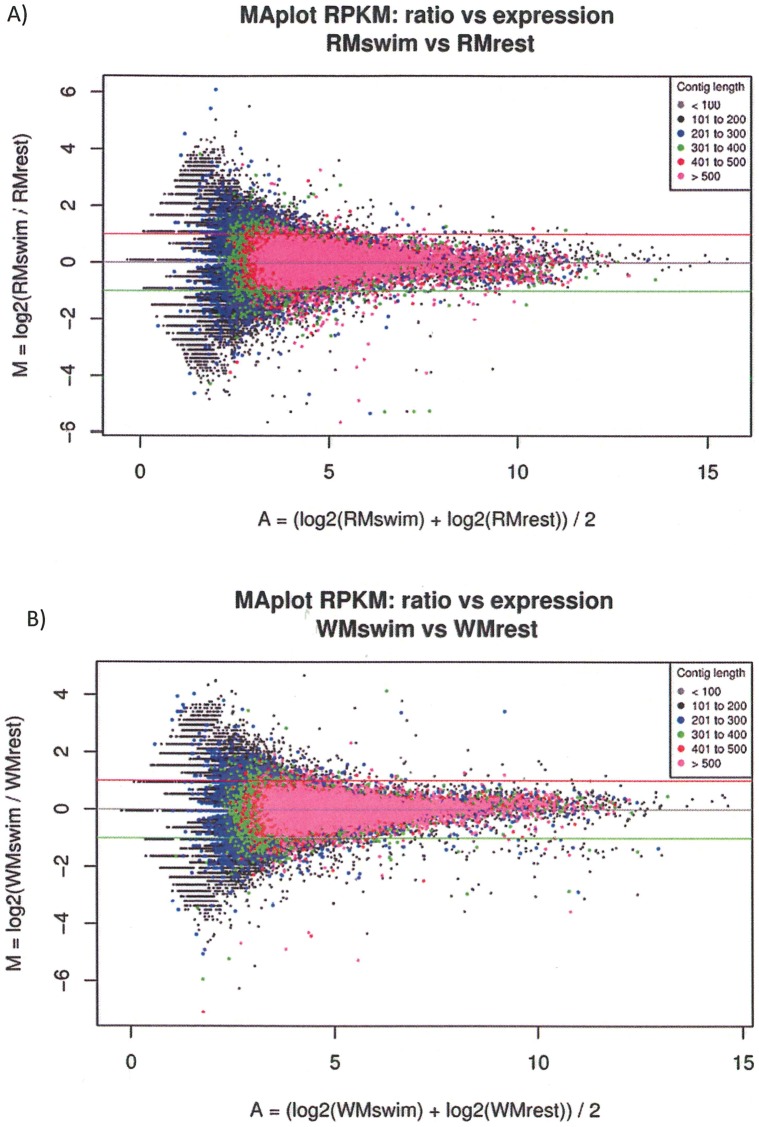
MA plots of the ratio vs. average expression by Reads Per Kilobase (exon model) per Million mapped reads (RPKM) value of the contigs as grouped in various size classes. A) red muscle and B) white muscle. The green and red lines indicate thresholds of differential expression at RPKM = 0.5 and RPKM = 2, respectively. Larger, more abundant contigs show less noise in differential expression as determined by RPKM.

Because RPKM values are determined on basis of 1000 nt, a decrease in contig size will result in increased noise (e.g. falsely determined differential expression). In order to visualize noise, MA plots were generated to display the ratio M = log2(RMrest/RMswim) vs. the average expression by RPKM A = (log2(RMswim)+log2(RMrest))/2 of the contigs as grouped in various sizeclasses (Fig. 1).

### Iterative Homology Search and Annotation

FASTA consensus sequences of the assembled contigs were annotated using an iterative homology search approach consisting of consecutive BLAST steps against different databases. After each BLAST step, XML results were analyzed with the Blast2GO pipeline (http://www.blast2go.org/: [10], [11]) using default settings and the best BLAST hit was recovered. Those contigs without or with uninformative descriptions were submitted to the next BLAST step; the others were considered to be well annotated and were kept. The different BLAST steps performed were 1) nucleotide BLAST (BLASTn or megaBLAST) against EST salmonid sequences of the SIGENAE database, the Information System of the AGENAE program (Analysis of Breeding Animals’ Genome) of INRA (http://www.sigenae.org/) with updated annotation (kindly provided by Dr. F.W. Goetz, Great Lakes WATER Institute University of Wisconsin-Milwaukee, Milwaukee, US); 2) BLASTx against the zebrafish *(Danio rerio)* Reference Sequence (refSeq) protein database and 3) BLASTx against the refSeq Metazoa protein database excluding *D. rerio*: The query ‘srcdb_refseq[prop] AND Metazoa[organism] NOT “*D. rerio*”[organism]’ was used in NCBI (http://www.ncbi.nlm.nih.gov/) to retrieve a “gi” list of non-*D. rerio* refSeqs and create a restricted alias of the refseq_protein database containing the refSeqs from all metazoa except *D. rerio.* Results from this step were not filtered for uninformative descriptions and they were all added to the final annotation file.

### Selection of Differentially Expressed Genes and RPKM Validation by Quantitative PCR (Q-PCR)

Differential expression as determined on the basis of RPKM values was validated by Q-PCR. Contigs were selected on the basis of stringent criteria. First, false differential expression noise due to size in differential expression as determined by RPKM values (Fig. 1) was minimised by focusing on contigs larger than 500 nt. Second, among these contigs, only those with SIGENAE salmonid annotation were selected. Other large contigs that were annotated by BLASTx vs. the zebrafish refSeq and refSeq Metazoa Proteins databases were considered novel gene sequences for rainbow trout. Third, only those contigs that were down-regulated at a fc ≤0.5 or up-regulated at a fc ≥2 were considered. Among these, contigs were selected with annotations that indicated key muscle functions. For validation by Q-PCR, 10 contigs for white muscle and 11 contigs for red muscle were selected. The contig sequences associated with the genes *guanylate-binding protein* (*gbp*) and *troponin T3b skeletal fast isoform 1* (*T3b1*) were similar for red muscle and white muscle. Primers were designed within the overlapping region of the two sequences for red muscle and white muscle and the same primers were used for both tissues. Other contig sequences were different for both tissues but were associated with the same genes, for example *troponin C skeletal muscle* (*tropC*), *IgM membrane heavy bound form* (*IgM*), *retinoic acid receptor gamma b* (*Rargb*) and *phosphofructokinase muscle b* (*pfkmb*). Furthermore, primers were designed for 4–5 contigs annotated as tissue specific differentially expressed genes. For red muscle, these were *follistatin-related protein 1* (*fstl1*), *myoblast determination protein 2* (*MyoD2*), *nuclear receptor coactivator 4* (*ncoa4*), *growth hormone 2* (*gh2*) and *fatty acid binding protein 6* (*fabp6*). For white muscle, these were *titin-like* (*ttn*), *four and a half LIM domains protein 1* (*fhl1*), *ubiquitin specific protease 14* (*ubp14*), and *heat shock protein 30* (*hsp30*). Primers to the targeted genes were designed using the Genamics Expression software (www.genamics.com) and given in Table S1.

For validation by qPCR, equal parts from each of the ten individual tissue samples per group were pooled. RNA was isolated with TRIzol (Invitrogen, Baro, Spain) and measured by Nanodrop, DNAse treated with RQ1 DNAse (Promega, Madison, USA) and reverse transcribed using Superscript III (Invitrogen, Baro, Spain), according to the manufacturers’ protocols. cDNA was diluted 1∶25 for target genes and 1∶2000 for *40S ribosomal protein S18* (*rps18*), and used as a template. The reactions (20 µl final volume) contained 10 µl of SYBR GreenER qPCR SuperMix (Invitrogen), 500 nM concentration of forward and reverse primers and 5 µl of cDNA. Reactions were run in a MyiQ Real-Time PCR Detection System (BioRad) using the following protocol: 2 min at 50°C, 8 min at 95 °C, followed by 40 cycles of 15 sec denaturation at 95°C and 30 sec at the corresponding melting temperatures, and a final melting curve of 81 cycles from 55°C to 95°C (0.5 °C increments every 10 sec). Samples were run in triplicate and fluorescence was measured at the end of every extension step. Fluorescence readings were used to estimate the values for the threshold cycles (Ct). The Ct values were normalized for each gene against those obtained for the housekeeping gene *rps18*. Normalized Ct values were expressed as fc using the relative quantification method [12], calculated for swimmers relative to resters. Fold changes on the basis of RPKM values and those determined by Q-PCR were compared for direction (up or down-regulated) and degree of expression.

### Gene Ontology (GO) Analysis

For each tissue, Blast2GO was used to create DAT files containing: 1) the best BLAST hits from the filtered BLASTn vs. salmonid SIGENAE database but with accession numbers changed from SIGENAE to their best BLAST hit protein id (ProtID) in order to be able to retrieve GeneOntology terms; 2) the best BLAST hits from the filtered BLASTx *vs.* the *D. rerio* refSeq database and 3) the best BLAST hits and non-annotated sequences from the BLASTx vs. refSeq Metazoa Proteins database excluding *D. rerio*. These files were used to perform GO analysis [10] on the whole transcriptome of red and white muscle. Specifically, both tissues were compared on biological processes, molecular functions and cellular components. A similar approach was followed in the identification of biological processes and molecular functions that were characterised by the involvement of differentially expressed contigs larger than 500 nt.

### Statistical Analyses

Two approaches were followed in this study. First, a comparison was made between the whole red and white muscle transcriptome for all contigs or only the large contigs. We used the package Gossip [13] for statistical assessment of annotation differences between both sets as integrated within Blast2GO. We tested differences between red and white muscle transcriptome using a two-tailed Fisher’s Exact Test that corrects for multiple testing providing a corrected p-value by False Discovery Rate (FDR) control. Red muscle was tested vs. white muscle as reference. Differences with FDR ≤0.05 were considered significant. Secondly, for quantifying the effects of exercise using RNA-seq, we pooled tissue samples of 10 fish per group as biological replicates. Statistical assessment of differences between the single values of the treatment groups was not possible but a generally accepted biologically meaningful cut-off level of fold change 2 was applied. The incorporation of biological replicates was at this stage of pioneering with RNA-seq applications for fish research still not affordable.

## Results

### RNA Sequencing (RNA-seq)

RNA-seq yielded 15.1 million (M) reads for white muscle in resters and 16.6 M reads in swimmers (Table 1). Yields in red muscle were higher than in white muscle, consisting of 17.9 M reads in resters and 17.4 M reads in swimmers (Table 1). *De novo* assembly per tissue and condition was performed at mapping efficiencies of reads into contigs of 42.1–45.3% for individual groups (Table 1). *De novo* assembly of combined resters and swimmers for each tissue yielded 149,159 contigs in red muscle and 118,572 in white muscle (Table 2). Maximal contig length was 15,779 nt in red muscle and 16,748 nt in white muscle. Many contigs were small and of lengths between 100 and 200 nt. In red muscle, 21.2% of the total number of contigs was ≥200 nt and 4.4% were ≥500 nt in length (corresponding to 6,512 contigs) (Table 2). Fewer but relatively longer contigs were found in white muscle: 27.0% of the total number of contigs was ≥200 nt and 5.0% were ≥500 nt in length (corresponding to 5,977 contigs) (Table 2).

**Table 1:** Summary statistics of Illumina sequencing.

	Red muscle		White muscle	
	Resters	Swimmers	Resters	Swimmers
Sequence reads (51 nt)	17.885,503	17.415,589	15.082,988	16.588,952
Mapped reads	8.097,376	7.642,409	6.743,174	6.992,219
unique	7.809,917	7.394,390	6.483,635	6.739,483
non-unique	287,459	248,019	259,539	252,736
Unmapped reads	9.788,127	9.773,180	8.339,814	9.596,733

The number of 51 nucleotides (nt) sequence reads, mapped reads (unique or non-unique) and unmapped reads for each individual group (red muscle or white muscle of resters or swimmers) are indicated.

**Table 2:** Results from *de novo* assembly and annotation per tissue (red or white muscle).

	RED MUSCLE		WHITE MUSCLE	
	Number	% of total	Number	% of total
**Total numbers**				
**Contigs (≥100 nt)**	149,159		118,572	
**Contigs ≥200 nt**	31,609	21.2	32,061	27.0
**Contigs ≥500 nt**	6,512	4.4	5,977	5.0
**Maximum contig length (nt)**	15,779		16,748	
**Number of contigs differentially expressed by RPKM**				
**Contigs down-regulated in swimmers vs. resters (fc ≤0.5)**	14,932	10.0	14,928	12.6
**Contigs up-regulated in swimmers vs. resters (fc ≥2)**	21,172	14.2	12,167	10.3
**Exercise specific contigs**	1,366	0.9	1,361	1.1
**Non exercise specific contigs**	1,503	1.0	1,185	1.0
**Contigs annotation accuracy and origin**				
**Sum of well annotated contigs**	66,035	44.3	61,437	51.8
**Annotation from SIGENAE salmonid ESTs**	46,785	31.4	41,696	35.2
**Annotation from zebrafish RefSeq proteins**	8,649	5.8	9,104	7.7
**Annotation from Metazoa RefSeq proteins**	10,601	7.1	10,639	9.0
**Contigs ≥500 nt**				
**Contigs down-regulated in swimmers vs. resters (fc ≤0.5)**	118	1.8	71	1.2
**Contigs up-regulated in swimmers vs. resters (fc ≥2)**	51	0.8	29	0.5

Numbers and maximum size of assembled contigs, differential contig expression by Reads Per Kilobase (exon model) per Million mapped reads (RPKM) values, iterative BLAST annotation results and differentially expressed contigs ≥500 nt are indicated.

### GO of Red and White Muscle Transcriptome

**Figure 2:**
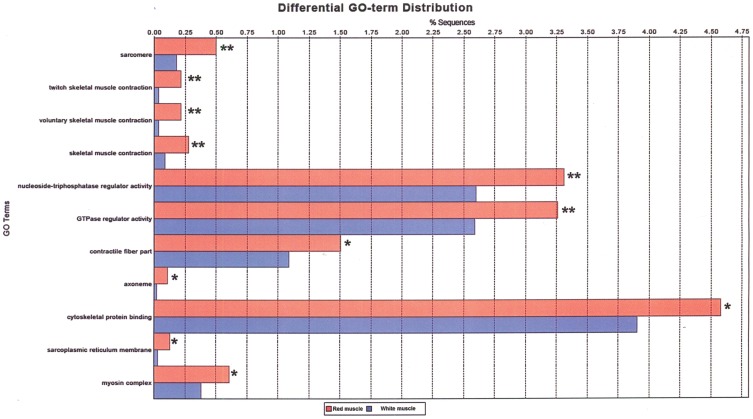
Differential GO term distribution between red and white muscle. The red muscle transcriptome (red bars) was tested against the white muscle transcriptome (blue bars). Significant differences by a corrected p-value for multiple testing False Discovery Rate (FDR) are indicated by ** FDR≤0.01 and * FDR≤0.05. Significant differential expression was found for biological processes including twitch skeletal muscle contraction, voluntary skeletal muscle contraction and skeletal muscle contraction; for molecular functions including nucleoside-triphosphatase regulator activity, GTPase regulator activity and cytoskeletal protein binding; and cellular components including sarcomere, contractile fiber part, axoneme, sarcoplasmic reticulum membrane and myosin complex. In all cases, the percentage of sequences was higher in red than in white muscle.

Visualizations of the main biological processes and molecular functions in the red and white muscle transcriptomes are provided in Figs. S1, S2, S3, S4. In terms of biological processes, the most abundant GO terms in both red and white muscle included transport, anatomical structure development, localization, nucleic acid metabolic process, signalling, cellular biosynthetic process and nitrogen compound metabolic process (Figs. S1 and S3). In terms of molecular functions, the most abundant GO terms in both red and white muscle included nucleic acid, protein and ion binding and hydrolase activity (Figs. S2 and S4). Testing the red muscle transcriptome against the white muscle transcriptome provided a differential GO term distribution between red and white muscle with significant differences by FDR (Fig. 2). Significant differential expression was found for biological processes such as those related to skeletal muscle contraction and cytoskeletal protein binding. Significant differential expression was found for molecular functions such as nucleoside-triphosphatase regulator activity and GTPase regulator activity. Finally, cellular components that were differentially expressed between red and white muscle were related to the sarcomere, the contractile fiber part, the axoneme, the sarcoplasmic reticulum membrane and the myosin complex. In all cases, the percentage of sequences was higher in red than in white muscle.

**Figure 3:**
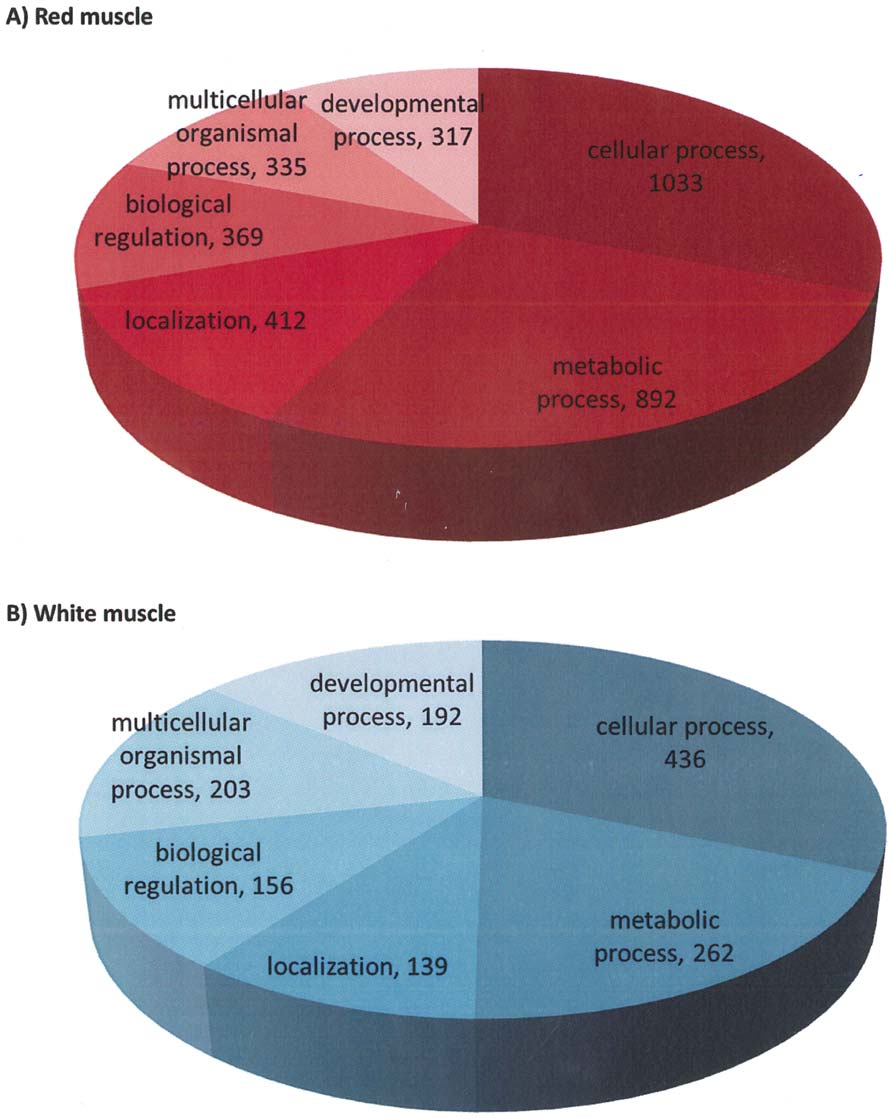
Gene ontology of the large contigs (≥500 nt) in red and white muscle. Illustrated as pie charts are the six largest categories in red muscle (A) and white muscle (B) at biological process level 2 with the numbers of sequences per category indicated. Gene ontology processes are cellular process; metabolic process; localization; biological regulation; multicellular organismal process and developmental process in both red and white muscle without any relevant percentual differences between both tissues. Categories are however represented by a larger number of sequences in red muscle than in white muscle.

GO of large contigs (≥500 nt) shows that contigs in red and white muscle are associated with the same six main categories: cellular process, metabolic process, localization, biological regulation, multicellular organismal process and developmental process (biological process level 2; Fig. 3). Similar to what was found when comparing the entire transcriptomes, GO categories of large contigs were represented by a much larger number of sequences in red muscle than in white muscle.

### Annotation and Identification of Novel Genes

The three-step iterative BLAST strategy resulted in 44.3% of the red muscle contigs and 51.8% of white muscle contigs being successfully annotated (Table 2). Most of them were megaBLAST hits obtained against the SIGENAE salmonid EST database, with 31.4% and 35.2% contigs annotated out of the total number of contigs in red and white muscle, respectively. Many small contigs were found exclusively in swimmers or in resters. The only longer contig (418 nt) that was specifically present in the white muscle of resters and not in that of swimmers was annotated as *interferon-induced very large GTPase 1-like*. Moreover, many other contigs in the white muscle of resters were annotated as this gene.

Interestingly, when the large contigs (≥500 nt) were blasted against the SIGENAE database, 4,627 red muscle (71.1%) and 4,303 white muscle (72.0%) contigs were annotated. The remaining large contigs were either annotated against the zebrafish refSeq and refSeq Metazoa protein databases and were considered novel rainbow trout sequences, or they could not be annotated. Thereby, we have identified 1,085 novel rainbow trout red muscle gene sequences associated with 811 unique gene names and 1,228 novel white muscle gene sequences associated with 928 unique gene names. In total, we have identified 1,432 novel rainbow trout transcripts. Most of these novel transcripts were tissue-specific and were associated with important functional properties of skeletal muscle, including key growth and myogenic factors, receptors, structural and cytoskeletal elements, signalling molecules, metabolic regulators, cell adhesion molecules and extracellular matrix components, ion channels and immune factors (Table 3; Table S2). Of all the novel sequences only 306 unique gene names were present in both red and white muscle.

**Table 3:** Selection of novel rainbow trout genes grouped by functional categories.

Putative Name and Function	Size, bp	Hit Acc. No.	E-Value	Blastx		Muscle
***Growth and Myogenic Factors***						
fibroblast growth factor 1 (acidic) [D. rerio]	659	NP_001098748	4.88E−31		Drerio	R
follistatin-like 1b [D. rerio]	539	NP_001034710	8.02E−89		Drerio	W
growth arrest-specific 7 [X laevis]	574	NP_001090555	4.02E−72		RefSeq	R
heparin-binding EGF-like growth factor [D. rerio]	1024	NP_001104696	5.52E−31		Drerio	R, W
insulin-like growth factor binding protein 5a [D. rerio]	565	NP_001119935	4.26E−43		Drerio	R
insulin-like growth factor binding protein 6b [D. rerio]	500	NP_001154874	3.11E−73		Drerio	W
insulin-like growth factor-binding protein 3 [B. taurus]	593	NP_776981	2.04E−37		RefSeq	W
myocyte enhancer factor 2ca [D. rerio]	600	NP_571387	1.23E−59		Drerio	R, W
myocyte-specific enhancer factor 2C [D. rerio]	634	NP_001124434	1.06E−35		Drerio	R
myogenic factor 6 [D. rerio]	562	NP_001003982	7.14E−67		Drerio	R, W
protein Wnt-2 [D. rerio]	2633	NP_878296	2.62E−128		Drerio	R
angiopoietin-1 [D. rerio]	1023	NP_571888	5.88E−57		Drerio	R, W
***Receptors***						
androgen receptor [D. rerio]	624	NP_001076592	1.38E−32		Drerio	W
cation-independent mannose-6-phosphate receptor [D. rerio]	1107	NP_001034716	3.81E−81		Drerio	W
TGF-beta receptor type-2 [D. rerio]	658	NP_878275	8.17E−95		Drerio	R
vascular endothelial growth factor receptor 1 [D. rerio]	536	NP_001014829	6.43E−11		Drerio	R
frizzled homolog 3-like [D. rerio]	512	NP_001074070	1.85E−52		Drerio	R
bone morphogenetic protein receptor, type 1a [D. rerio]	661	NP_571696	6.21E−39		Drerio	R
leukemia inhibitory factor receptor alpha [D. rerio]	1278	NP_001014328	2.68E−89		Drerio	W
ryanodine receptor 1b (skeletal) [D. rerio]	3716	NP_001096041	0.0		Drerio	R, W
ryanodine receptor 3 [G. gallus]	845	NP_996757	2.46E−111		RefSeq	R, W
acetylcholine receptor subunit alpha [D. rerio]	1860	NP_571520	0.0		Drerio	R, W
acetylcholine receptor subunit beta [M. musculus]	520	NP_033731	2.60E−50		RefSeq	W
acetylcholine receptor subunit delta [D. rerio]	606	NP_996947	2.40E−18		Drerio	R, W
***Structural and Cytoskeletal Elements***						
myosin heavy chain, cardiac muscle isoform [G. gallus]	603	NP_990097	5.02E−52		RefSeq	R
myosin-binding protein C, slow-type [D. rerio]	782	NP_001007323	1.21E−111		Drerio	R
myosin-Va [D. rerio]	1990	NP_001074428	0		Drerio	R
myosin-VI [D. rerio]	500	NP_001004110	2.24E−68		Drerio	R
dystrophin [D. rerio]	624	NP_571860	7.21E−18		Drerio	R
actin-binding protein IPP [D. rerio]	1013	NP_001107093	1.83E−02		Drerio	W
actin-binding Rho-activating protein [D. rerio]	805	NP_001003986	1.10E−74		Drerio	W
actin-related protein 3B [D. rerio]	780	NP_001083025	1.76E−113		Drerio	W
alpha-actinin-2 [D. rerio]	1369	NP_001032662	0		Drerio	R
ankyrin 1, erythrocytic [D. rerio]	643	NP_001005969	9.70E−21		Drerio	R, W
ankyrin 3, epithelial isoform 1 [R. norvegicus]	1195	NP_113993	1.70E−04		RefSeq	W
huntingtin [D. rerio]	580	NP_571093	4.42E−91		Drerio	R
supervillin [D. rerio]	1705	NP_001030338	0.0		Drerio	R, W
synaptopodin-2 [R. norvegicus]	513	NP_001178892	1.14E−23		RefSeq	R
synemin [D. rerio]	655	NP_001038340	1.40E−46		Drerio	R, W
tensin 1 [R. norvegicus]	603	NP_001178739	6.56E−48		RefSeq	W
troponin I, skeletal, slow like [D. rerio]	696	NP_001002101	2.83E−45		Drerio	R
troponin T type 1 (skeletal, slow) [X laevis]	1067	NP_001086207	3.06E−67		RefSeq	R
troponin T2a, cardiac [D. rerio]	891	NP_690853	5.36E−61		Drerio	R
***Protein Kinases and Phosphatases***						
5′-AMP-activated protein kinase alpha-2 [G. gallus]	613	NP_001034694	1.83E−89		RefSeq	R
mitogen-activated protein kinase kinase kinase 5 [D. rerio]	764	NP_001155222	1.82E−107		Drerio	R, W
protein kinase C, theta [D. rerio]	667	NP_001082839	4.76E−90		Drerio	W
striated muscle preferentially expressed protein kinase [D. rerio]	2086	NP_001007110	1.10E−141		Drerio	R, W
protein phosphatase 1 regulatory subunit 3C [D. rerio]	616	NP_957128	7.79E−81		Drerio	R, W
protein phosphatase 1L [D. rerio]	1119	NP_001071068	0.0		Drerio	W
protein phosphatase inhibitor 2 [G. gallus]	723	NP_001026484	1.17E−06		RefSeq	W
Serine/threonine-protein phosphatase 2B catalytic subunit alpha isoform [S. salar]	649	NP_001135322	4.11E−122		RefSeq	R
serine/threonine-protein phosphatase 5 [D. rerio]	1291	NP_001014372	0.0		Drerio	R, W
hepatocyte growth factor-regulated tyrosine kinase substrate [D. rerio]	725	NP_956162	1.62E−119		Drerio	W
***Metabolism***						
6-phosphofructo-2-kinase/fructose-2,6-biphosphatase 1 [D. rerio]	772	NP_956102	1.09E−104		Drerio	R
brain creatine kinase [D. rerio]	737	NP_001070631	4.68E−04		Drerio	R, W
acetyl-CoA carboxylase beta [X. tropicalis]	1947	NP_001131086	0		RefSeq	R
acetyl-coenzyme A synthetase, cytoplasmic [D. rerio]	575	NP_001002641	1.15E−59		Drerio	W
glycogen phosphorylase, muscle form [D. rerio]	568	NP_001018464	6.92E−99		Drerio	R
glycogenin 1 [D. rerio]	509	NP_998675	3.52E−85		Drerio	R
mitochondrial uncoupling protein 2 [D. rerio]	1737	NP_571251	5.97E−130		Drerio	R, W
phosphoglycerate kinase 1 [D. rerio]	690	NP_998552	3.05E−79		Drerio	W
phosphorylase b kinase regulatory subunit alpha, skeletal muscle isoform [R. norvegicus]	634	NP_072148	2.67E−41		RefSeq	W
***Cell adhesion/Extracellular Matrix***						
laminin subunit alpha-1 [D. rerio]	3762	NP_001030158	0.0		Drerio	W
laminin subunit beta-1 [D. rerio]	752	NP_775382	5.48E−40		Drerio	R, W
integrin alpha-6 [D. rerio]	1086	NP_001138253	2.65E−96		Drerio	W
integrin, beta 1a [D. rerio]	551	NP_001030143	5.60E−61		Drerio	R, W
collagen type IV alpha1 [O. latipes]	657	NP_001170943	8.53E−14		RefSeq	R
collagen type IX alpha I [D. rerio]	1653	NP_998429	8.32E−49		Drerio	R, W
collagen, type IV, alpha 2 [G. gallus]	569	NP_001155862	1.51E−63		RefSeq	R, W
collagen, type XI, alpha 1 [D. rerio]	1718	NP_001077313	2.05E−90		Drerio	R, W
basement membrane-specific heparan sulfate proteoglycan core protein [D. rerio]	521	NP_001120939	7.01E−71		Drerio	R, W
A disintegrin and metalloproteinase with thrombospondin motifs 10 [D. rerio]	582	NP_001116746	1.70E−99		Drerio	R
***Ion channels***						
potassium voltage-gated channel subfamily A member 6 [D. rerio]	1103	NP_001124098	5.48E−81		Drerio	W
sodium- and chloride-dependent creatine transporter 1 isoform 3 [M. musculus]	1370	NP_001136282	1.15E−155		RefSeq	W
sodium channel protein type 4 subunit alpha A [D. rerio]	753	NP_001034914	7.36E−77		Drerio	W
sodium channel, voltage gated, type XII, alpha a [D. rerio]	707	NP_001038387	1.15E−110		Drerio	R
sodium channel, voltage-gated, type IV, beta a [D. rerio]	522	NP_001071038	1.54E−57		Drerio	W
voltage-dependent calcium channel gamma-6 subunit [D. rerio]	650	NP_001076560	3.54E−18		Drerio	R, W
voltage-dependent calcium channel subunit alpha-2/delta-1 [D. rerio]	920	NP_001038425	4.99E−110		Drerio	R
calcium channel, voltage-dependent, L type, alpha 1S subunit, a [D. rerio]	2299	NP_001139622	0.0		Drerio	R, W
***Protein degradation***						
E3 ubiquitin/ISG15 ligase TRIM25 [B. taurus]	538	NP_001093806	1.68E−07		RefSeq	R
E3 ubiquitin-protein ligase HECTD1 [D. rerio]	582	NP_001002504	1.20E−11		Drerio	W
E3 ubiquitin-protein ligase MARCH5 [D. rerio]	730	NP_956033	5.41E−37		Drerio	R, W
E3 ubiquitin-protein ligase mib1 [D. rerio]	506	NP_775393	3.30E−86		Drerio	W
E3 ubiquitin-protein ligase NEDD4 [D. rerio]	770	NP_001029358	6.72E−126		Drerio	W
E3 ubiquitin-protein ligase RING2 [D. rerio]	727	NP_571288	2.21E−107		Drerio	W
E3 ubiquitin-protein ligase SMURF1 [D. rerio]	726	NP_001001943	2.12E−110		Drerio	W
E3 ubiquitin-protein ligase TRIM32 [D. rerio]	873	NP_001107066	1.94E−10		Drerio	W
ubiquilin-4 [D. rerio]	600	NP_998521	7.89E−22		Drerio	W
ubiquitin carboxyl-terminal hydrolase 25 [D. rerio]	1575	NP_001001886	0.0		Drerio	R, W
ubiquitin specific peptidase 47 [R. norvegicus]	610	NP_001101012	5.83E−80		RefSeq	R
ubiquitin specific peptidase 7 (herpes virus-associated) [X. laevis]	537	NP_001121282	6.28E−58		RefSeq	W
ubiquitin-associated protein 2-like [D. rerio]	520	NP_001076535	1.37E−50		Drerio	W
ubiquitin-conjugating enzyme E2 E3 [D. rerio]	598	NP_957215	1.21E−14		Drerio	R, W
ubiquitin-like modifier-activating enzyme 1 [D. rerio]	815	NP_998227	1.34E−127		Drerio	W
ubiquitin-protein ligase E3A [D. rerio]	1101	NP_001007319	2.68E−18		Drerio	W
26S proteasome non-ATPase regulatory subunit 2 [D. rerio]	523	NP_956840	2.60E−81		Drerio	W
26S proteasome non-ATPase regulatory subunit 4 [M. domestica]	797	NP_001130032	2.54E−22		RefSeq	W
calpain 1, (mu/I) large subunit a [D. rerio]	779	NP_956739	4.42E−142		Drerio	R, W
Calpain-3 [S. salar]	2559	NP_001158880	0.0		RefSeq	W
cathepsin Z [X laevis]	500	NP_001088101	4.86E−48		RefSeq	W
cationic trypsin-3 [D. rerio]	509	NP_571783	6.69E−55		Drerio	W
***Immune Factors***						
interferon regulatory factor 4 [D. rerio]	1202	NP_001116182	5.61E−46		Drerio	R
leukocyte receptor cluster member 8 homolog [D. rerio]	820	NP_001082988	4.38E−118		Drerio	R, W
NF-kappa-B inhibitor epsilon [D. rerio]	539	NP_001073558	8.99E−40		Drerio	W
nuclear factor NF-kappa-B p100 subunit [S. salar]	1084	NP_001167054	2.68E−130		RefSeq	W
scavenger receptor class B, member 2 [D. rerio]	1631	NP_775366	9.74E−135		Drerio	R, W
signal transducer and activator of transcription 2 [S. salar]	1072	NP_001138896	3.69E−124		RefSeq	W
signal transducer and activator of transcription 5.1 [D. rerio]	1168	NP_919368	0.0		Drerio	W
thymosin beta [D. rerio]	574	NP_991144	1.17E−03		Drerio	R, W

### Differential Gene Expression in Skeletal Muscle in Response to Exercise

Among all contigs in red muscle, 10.0% were down-regulated at fc ≤0.5 and 14.2% were up-regulated at fc ≥2 (Table 2) in swimmers. Similar values were obtained in white muscle of swimmers, with 12.6% of the contigs down-regulated at fc ≤0.5 and 10.3% up-regulated at fc ≥2. In red muscle, 1,366 contigs appeared in swimmers only and thus indicated exercise specific transcripts, while 1,503 contigs appeared in resters only (Table 2). In white muscle, 1,361 contigs appeared in swimmers only and 1,185 contigs appeared in resters only. However, all of these contigs that were specific for swimmers or resters were small (≤300 nt) except for the mentioned interferon-induced very large GTPase 1-like contig of 418 nt with a RPKM value of 20.2 that was only found in the white muscle of resters. Because RPKM values are determined on basis of 1000 nt, decreases in contig size are associated with increased noise (Fig. 1), which can be defined as the occurrence of false differentially expressed contigs due to their small size. Because of this, contig size thresholds need to be applied for justifying the quantification of expression. In the present study, although abundance was positively correlated to size, as also reported by Hegedus et al. [14], we have applied a size threshold of ≥500 nt since this size group was virtually without noise as visualized by MA plots (Fig. 1). Contigs that were smaller than 500 nt showed increasing false differential expression noise with smaller size (Fig. 1). The larger contigs (≥500 nt) showed less noise in differential expression as determined by RPKM. In red muscle, the expression of 118 large contigs was down-regulated at fc ≤0.5 (Table S3) and 51 were up-regulated at fc ≥2 in swimmers (Table S4). In the white muscle of swimmers, the expression of 71 large contigs was down-regulated at fc ≤0.5 (Table S5) and 29 were up-regulated at fc ≥2 (Table S6).

### Differentially Expressed Genes Common in Red and White Muscle

Red and white muscle shared only seven contigs that were differentially expressed in response to exercise on the basis of RPKM values. The expression of four of these contigs was different between the two tissues in response to exercise: *pfkb*, *Rargb*, *tropC* and *tropT3b1*. The expression of the other contigs was similar for both tissues: *gbp* and *IgM* (up-regulated) and *thymosin beta* (down-regulated). In addition to *tropC* and *tropT3b1*, other troponins were found differentially expressed in both muscle types, including *troponin I, slow skeletal muscle* [*Anoplopoma fimbria*], *troponin T type 1 (skeletal, slow)* [*Xenopus laevis*] and *troponin T2a, cardiac* [*D. rerio*] (Tables S3, S4, S5, S6). Other contig groups showed a marked tissue specificity in their differential expression. For instance, 37 contigs associated with *titin* were only differentially expressed in white muscle and were down-regulated. Similarly, eight contigs associated with myosin were only differentially expressed in red muscle and were down-regulated except for *myosin-7B* [*Gallus gallus*] that was up-regulated.

### Q-PCR Validation of RPKM Fold Changes

Among the annotated large contigs (≥500 nt) that showed differential expression on the basis of RPKM values, 10–11 contigs were selected per tissue for validation by Q-PCR (Table S7). Among those selected contigs that were differentially expressed in the red muscle of swimmers, 5 contigs were up-regulated and 6 contigs were down-regulated. In this tissue, the direction of expression was confirmed by Q-PCR for 7 contigs: 5 up-regulated (*gbp*, *IgM*, *pfkmb*, *ncoa4, gh2*) and 2 down-regulated (*fstl1*, *fabp6*); whereas the expression of 3 contigs (*tropT3b1, tropC, Myod2)* was different between the two methods (Table S7). In white muscle of swimmers, of the 10 differentially expressed contigs that were selected (6 up-regulated and 4 down-regulated), the direction of expression was confirmed by Q-PCR for 7 contigs: 4 up-regulated (*gbp*, *tropT3b1, tropC, fhl1*) and 3 down-regulated (*pfkmb ttn, ubp14*); whereas the expression of 3 contigs (IgM, *Rargb*, *hsp30*) was different between the two methods (Table S7).

## Discussion

In this study, we have performed RNA-seq to provide an in-depth view, for the first time, of the transcriptome of skeletal muscle of the rainbow trout, a commercially important species still without a sequenced genome. In particular, we have sequenced and compared the red and white muscle transcriptome and have used RNA-seq for the quantification of the effects of exercise in the rainbow trout skeletal muscle. Importantly, novel rainbow trout gene sequences have been identified in this study: 1,085 gene sequences in red muscle and 1,228 gene sequences in white muscle.

### Applying RNA-seq to Identify Genes Expressed in Rainbow Trout Skeletal Muscle and to Measure Changes in the Red and White Muscle Transcriptomes in Response to Exercise

*De novo* assembly of reads into contigs per tissue was performed at efficiencies between 42.1 and 45.3%. Efficiencies were thus rather low and more than half of the reads had to be discarded. Most contigs were small in the absence of a sequenced rainbow trout genome (Table 2), with 79% of the red muscle contigs and 73% of the white muscle contigs having lengths between 100 and 200 nt.

Annotation efficiencies obtained by the three-step iterative homology search approach used in this study were 44.3% for the red muscle contigs and 51.8% for the white muscle contigs. These annotation efficiencies were acceptable, especially when taking into account the absence of a sequenced trout genome, and are comparable to other RNA-seq transcriptomic studies [15], [16], [17]. The SIGENAE salmonid EST database provided ∼70% of the BLAST hits. Annotation efficiencies of the large contigs (≥500 nt) (6,512 contigs in red muscle and 5,977 contigs in white muscle) were ∼70% and approached those reported for zebrafish [18].

One of the most important findings of this study is the identification of a number of novel rainbow trout gene sequences in red and in white muscle by a homology search strategy against the zebrafish (*D. rerio*) genome and general Metazoan genes. In total, we have identified 1,432 novel rainbow trout sequences but among these, however, there were many hypothetical, probable and predicted protein sequences. When these unannotated sequences were filtered out of the results, 731 novel rainbow trout annotated sequences remained. Among this novel set of rainbow trout sequences (Table 3; Table S2), we have identified important growth and myogenic factors and their receptors that participate in the regulation of myogenic proliferation and differentiation, such as myocyte enhancer factor 2C (MEF2C), myogenic factor 6 (Myf6, also known as muscle-specific regulatory factor 4), fibroblast growth factor 1 (acidic), follistatin-like 1b, heparin-binding EGF-like growth factor, TGF-beta receptor type-2, bone morphogenetic protein receptor, type 1a and leukemia inhibitory factor receptor alpha [19]–[26]. Of particular interest is the identification of Wnt-2 and its receptor Frizzled homolog 3-like, components of the Wnt pathway that has been associated with skeletal muscle development and with adult muscle hypertrophy induced by mechanical overload in mammals [27], [28]. In addition, components of the insulin-like growth factor (IGF) family were identified, including the cation-independent mannose-6-phosphate receptor or IGF-2 receptor, known to transduce the myogenic differentiation-promoting effects of IGF-2 in muscle [29], and several IGF binding proteins (IGFBP3, 5 and 6b) that regulate the biological activity of IGF-1 [30]. Interestingly, there is also evidence for a possible role of androgens in trout skeletal muscle function as inferred from the identification of the androgen receptor (AR) as well as from genes that increase AR expression, such as supervillin, a cytoskeletal protein that is also a myogenic regulator [31], and the signal transducer and activator of transcription 5 (STAT5) [32]. Another member of the STAT family identified in our study is STAT2, known to promote myogenic differentiation by increasing the expression of myogenic factors and IGF-2 [33]. Apart from a number of novel structural and cytoskeletal elements (e.g. myosins, troponins and alpha-actinins), cell adhesion and extracellular matrix components (e.g. laminin, integrin and collagens) and ion channels (voltage-dependent calcium, sodium and potassium channels), we have identified ryanodine and acetylcholine receptors, known to be involved in the neural regulation of muscle activity (reviewed in [16] and [24]), and two forms of ankyrin (ankyrin 1 or ankyrin-R and ankyrin 3 or ankyrin-G) that play key roles in the assembly and functioning of membrane domains in skeletal muscle fibers [34], [35]. Not surprisingly, several key metabolic regulators were also identified in trout skeletal muscle, including genes involved in carbohydrate metabolism (glycogen phosphorylase, glycogenin, 6-phosphofructo-2-kinase, phosphorylase b kinase regulatory subunit alpha, etc.), AMP-activated protein kinase (AMPK) alpha-2, a well-known “fuel gauge” that transduces the effects of exercise-induced muscle contraction [36], protein kinase C theta and, importantly, mitochondrial uncoupling protein 2, a known regulator of energy metabolism [37] that is known to be up-regulated in response to contractile activity in mammals [38]. Finally, we identified a large set of genes involved in the regulation of protein degradation, mostly belonging to the ubiquitin-proteasome pathway, but also calpain proteases 1 and 3, with known roles in myofibrillar protein turnover (reviewed in [39]), and NF-κB p100 subunit and NF-κB inhibitor epsilon, two signalling molecules acting downstream of the action of tumor necrosis factor α or TNFα), a pro-inflammatory cytokine that contributes to muscle atrophy in mammals [40]. Although just a few of the novel genes reported here have been identified in other teleost species, the identified genes and the pathways in which they participate are expected to play an important role in skeletal muscle function in teleost fish (reviewed in [41]). In support of this idea, AMPK has recently been shown by our group to stimulate glucose uptake through a GLUT4-mediated mechanism and to increase the mRNA levels of genes involved in glucose metabolism (including 6-phosphofructo-2-kinase) in trout muscle cells in primary culture [42]. Furthermore, we have also recently shown that NF-κB is an important intracellular signalling molecule mediating the stimulatory effects of TNFα on glucose uptake in muscle cells [43].

In teleost fish, red and white skeletal muscle types are believed to play different but complementary roles during swimming. Whereas red muscle mainly powers sustained swimming, white muscle is involved mostly in burst swimming or sprinting [1]. Under the conditions of the present study, fish swam at near their optimal swimming speed and, therefore, a higher implication of the red muscle in swimming was expected. Our results suggest that red and white muscle of rainbow trout may differ in terms of their transcriptomic characteristics. First, the red muscle transcriptome appeared to be slightly larger than the white muscle transcriptome in terms of the number of reads (Table 1) and contigs (Table 2), although these small differences were not assessed statistically. Second, all differential GO terms between the two muscle transcriptomes, when considering only the large contigs (≥500 nt), appeared to score higher in number of sequences for red muscle than for white muscle (Fig. 2) and reflected the biological processes, molecular functions and cellular components involved in muscle contraction. Furthermore, large contigs associated with biological processes were more abundant in red muscle than in white muscle. Significant white muscle contribution to exercise is suggested, however, by the observations that the white muscle transcriptome of swimmers appeared to be 10% larger in terms of number of reads than that of resters (Table 1) and that the red muscle transcriptome was smaller in swimmers than in resters. Further support for the transcriptomic differences between red and white skeletal muscle in rainbow trout comes from our observation that genes involved in the response to anabolic androgens (AR and STAT5) and in protein degradation (NF-κB and ubiquitin-proteasome pathways, proteolytic enzymes) were almost exclusively expressed in white skeletal muscle (Table 3), in which the interplay between protein synthesis and degradation appears to be more relevant. Furthermore, troponin T3b (fast isoform 1) and troponin C, two key regulators of skeletal muscle contraction, were only up-regulated in white muscle of swimmers, as confirmed by Q-PCR. On the other hand, all three slow troponins identified in this study (troponin I, troponin T1 and Troponin T2a) were expressed only in red muscle, in accordance with the particular biochemical and contractile characteristics of this type of muscle. Interestingly, *fhl1*, a gene known to promote hypertrophy in skeletal muscle in mammals [44], was clearly up-regulated in white muscle of swimmers, as confirmed by Q-PCR. Specific for the red muscle, however, was the up-regulation of *ncoa4*, a gene believed to be involved in muscle hypertrophy through its stimulation of protein synthesis [45]. It is worth mentioning that MYH7b, a gene that in mammals encodes the intronic miR-499, was found to be up-regulated in the red muscle of swimmers. In mammals, expression of miR-499 drives the conversion of fast myofibers to slow, type I myofibers in skeletal muscle [46] and, in view of this, it is tempting to speculate that swimming-induced contraction in trout may have increased the aerobic capacity of red skeletal muscle. Overall, our results show that muscle developmental processes appeared to be up-regulated in white muscle, but in red muscle the expression of genes involved in muscle development was either up-regulated or down-regulated. These results suggest that the anorexic swimming performance of simulated reproductive migration in rainbow trout may contribute to muscle growth and development, at least for the white muscle, supporting a role for this tissue in sustained swimming, and, thus, provide molecular support to the known stimulation of muscle fiber hypertrophy by swimming-induced activity in fish (reviewed in [47]). Further studies investigating the relationship between swimming-induced changes in the skeletal muscle transcriptome and the morphometric characteristics of muscle fibres will be necessary to understand the molecular basis of the growth-potentiating effects of swimming in fish.

Our results on the validation by qPCR of differentially expressed contigs by RNAseq in white and red skeletal muscle of exercised fish showed a 70% agreement with the two methods. However, the remaining contigs did not show the same direction in expression difference in response to exercise when the two methods were compared. We attribute these differences in gene expression changes between RNAseq and qPCR, first and most importantly, to the fact that contigs were generated by *de novo* assembly and not by use of a reference genome, since the trout genome is not available yet, and, second, to the differences in dynamic range between these two methods [8].

### The Migration Phenotype: Muscle Development and Steroid Binding of the Muscle

In this study, we have mimicked the conditions experienced by trout during reproductive migration with the specific aim to provide insight into the relationship between migration and sexual maturation, two seemingly competing processes representing a state of muscle building versus a state of muscle wasting, respectively [4]–[6], [48]. In this regard, we hypothesize that sex steroids, and particularly androgens, may play a key role in exercise-enhanced muscle building. Support for this hypothesis may come from our observations that AR and STAT5, a regulator in turn of the expression of AR [32], are expressed exclusively in white muscle and that *ncoa4*, believed to exert its hypertrophic action by interacting with AR and enhancing its activity [45], is up-regulated in red muscle of swimmers. Interestingly, the hypertrophy-promoting gene *fhl1* [44], up-regulated in white muscle of swimmers, is known to be involved in the transcriptional regulation of estrogen signalling [49] and its transcription in mammalian skeletal muscle cells is inhibited by estrogens [50]. This suggests that, in contrast to androgen-response mechanisms, estrogen-responsive mechanisms may not be active in white muscle of exercised rainbow trout. In support for the notion that androgens could be involved in the acquisition of the migration phenotype and that estrogens may antagonize it, a recent study has shown that testosterone may exert growth-promoting effects due to its ability to directly sensitize the white muscle to the effects of growth hormone (GH) and IGF-1 whereas 17β-estradiol (E_2_) attenuates the growth-promoting effects of GH and IGF-1 [51].

### The Sexual Maturation Phenotype: Interaction of Skeletal Muscle with the Reproductive Axis

Several clues regarding the interaction between skeletal muscle and the reproductive axis may provide more insight into the progression of the sexual maturation phenotype. As indicated above, estrogens may play an important regulatory role in muscle building but also have important reproductive roles. In fish, E_2_ is produced by the granulosa cells of the ovary during gonadal growth and induces the hepatic production of vitellogenin (vtg; reviewed in [52]). E_2_ may be the most important indicator of energy re-partitioning to ensure reproductive success [53]. Extrahepatic expression of vtg is known to occur in adipocytes present in various tissues, but generally at lower levels (<10%) than those in the liver, as shown in zebrafish [54]. In this study, we found several contigs associated with the *vtg1* gene expressed in red and white muscle. Interestingly, all the *vtg* contigs found in white muscle (5 small and 1 large) were expressed either exclusively or predominantly in resters (Table S8), suggesting that *vtg* expression in white muscle may be down-regulated in this tissue in response to swimming. Although it is not known if *vtg* expression in trout skeletal muscle can be regulated by E_2_, we hypothesize that the down-regulation of *vtg* expression in white muscle of swimmers could be related to a possible down-regulation of estrogen-responsiveness that could characterize the migration phenotype, as stated above. In fact, several small contigs for estrogen receptor beta were found in white and red muscle of trout although without apparent differences in expression between resters and swimmers (Table S8).

On the other hand, in white muscle a small contig was annotated as the *kiss1 receptor* (*kiss1r*) (Table S8) and we hypothesize that it may play a role in receiving maturity signals by circulating kisspeptin. Recent studies on the initiation of puberty in mammals have revealed a crucial role of the Kiss1/Kiss1 receptor pathway in this process [55] and there is evidence for a similar role in fishes [56], [57]. Kisspeptin acts as a somatotropic messenger [58] on the hypothalamic-pituitary-gonadal axis in mammals. Kisspeptin signals the hypothalamus to release Gonadotropin Releasing-Hormone [56] or may even act directly on the pituitary, as suggested by our observation on the expression of *kiss1r* in the pituitary of rainbow trout when stimulated under reproductive conditions [4]. As a somatotropic messenger, it is possible that kisspeptin may also act on the rainbow trout white muscle and elucidation of the possible actions of kisspeptin on white skeletal muscle would deserve further study.

Furthermore, a potential signal to the reproductive axis originating in skeletal muscle may be follicle stimulating hormone (FSH). We found expression of the beta subunit of FSH (*fshb*) in red and white muscle, with 6 contigs corresponding to *fshb* in red muscle and three in white muscle, although most did not show important changes in expression in response to swimming (Table S8). Pituitary FSH stimulates ovarian E_2_ production and Vtg uptake by the oocyte [52], [59], [60] and it is tempting to speculate that muscle-derived FSH could be involved in the switch from previtellogenic oocytes of the migration phenotype to the vitellogenic oocytes of the sexual maturation phenotype. It would be very interesting to determine the circulating FSH levels and quantify the ‘classic’ contribution of the pituitary and the potential contribution of the skeletal muscle.

### Conclusions

By performing deep RNA-seq of red and white skeletal muscle in rainbow trout we have been able to catalogue the transcriptome and identify differences in gene expression between both types of muscles. Importantly, a number of novel rainbow trout gene sequences have been identified in this study: 1,085 and 1,228 novel gene sequences for red and white muscle, respectively, that include a number of important molecules for skeletal muscle function. White muscle shows up-regulated muscle developmental processes and increased activity in swimmers. Furthermore, indirect evidence suggests that gonadal steroids may play an important regulatory role in the development of the migration phenotype, as characterized by aerobic muscle contraction. In turn, the skeletal muscle may interact with the reproductive axis possibly through its binding of gonadal steroids and Kisspeptin, and through its production of Vtg and FSH.

### Perspectives

The unique collection of transcript sequences in red and white muscle that was obtained in this study is a valuable dataset that will contribute to our understanding of red and white muscle functioning in relation to swimming-induced activity, and specifically during long-term salmonid reproductive migration. Moreover, *de novo* assembly on the reads of all four groups together will contribute to improve the coverage of the rainbow trout red and white muscle transcriptomes. This dataset will be important for the identification and characterization of essential genes regulating processes involved in the response of skeletal muscle to swimming-induced activity in teleost fish.

## Supporting Information

Figure S1 **Sequence distribution of GO terms in relation to the number of sequences (SeqNr) of biological processes of the red muscle transcriptome.**
(TIF)

Figure S2 **Sequence distribution of GO terms in relation to the number of sequences (SeqNr) of molecular functions of the red muscle transcriptome.**
(TIF)

Figure S3 **Sequence distribution of GO terms in relation to the number of sequences (SeqNr) of biological processes of the white muscle transcriptome.**
(TIF)

Figure S4 **Sequence distribution of GO terms in relation to the number of sequences (SeqNr) of molecular functions of the white muscle transcriptome.**
(TIF)

Table S1 **Nucleotide sequence of primers used for validation of selected target genes by Q-PCR.** Forward (F) and reverse (R) primers were designed for the housekeeping gene rps18 (Genbank accession number AF308735) and for target genes on basis of the nucleotide sequence of selected contigs that were larger than 500 nt, that had a SIGENAE salmonid annotation and that were differentially expressed at a fc ≤0.5 or fc ≥2. Primers were designed on basis of the overlapping region between the two sequences for red muscle (R) and white muscle (W) and the same primers were used for both tissues (‘R, W’), on basis of contig sequences that were different for both tissues but were associated with the same genes (‘R’ and ‘W’), or on basis of contig sequences that were tissue specific differentially expressed (‘R’ or ‘W’).
(DOCX)

Table S2 **List of novel rainbow trout genes identified by RNAseq.** Genes expressed in red and white skeletal muscle are listed according to sequence length. Putative name, length of sequence (Size, in bp), hit accession number, E-value and database where sequence was identified by BLASTx are indicated.
(XLSX)

Table S3 **Down regulated contigs (>500 nt) in the red muscle of swimmers.** Columns show the number of the specific contig, its annotation, the database from which a BLAST hit was obtained (SIGENAE salmonids, Refseq zebrafish, Refseq metazoa), the length of the contig in nucleotides, the Reads Per Kilobase (exon model) per Million mapped reads (RPKM) value of swimmers, the RPKM value of resters and the fold change of swimmers vs. resters.
(DOCX)

Table S4 **Up regulated contigs (>500 nt) in the red muscle of swimmers.** *See legend Table S3 for a description.*
(DOCX)

Table S5 **Down regulated contigs (>500 nt) in the white muscle of swimmers.** *See legend Table S3 for a description.*
(DOCX)

Table S6 **Up regulated contigs (>500 nt) in the white muscle of swimmers.** *See legend Table S3 for a description.*
(DOCX)

Table S7 **Q-PCR validation of fold change (fc) expression by read-per-kilo-base-of-exon-model (RPKM) values of selected contigs in red and white muscle of swimmers.** Contigs were selected based on whether they were larger than 500 nt, had a SIGENAE salmonid annotation and were differentially expressed at a fc ≤0.5 or fc ≥2. Columns represent contig annotation, sequence length of the contig, fold change (fc) expression of swimmers over resters by RPKM and by Q-PCR. Abbreviations: *guanylate-binding protein* (*gbp*); *troponin T3b skeletal fast isoform 1* (*T3b1*); *troponin C skeletal muscle* (*tropC*); *IgM membrane heavy bound form* (*IgM*); *retinoic acid receptor gamma b* (*Rargb*); *phosphofructokinase muscle b* (*pfkmb*); *follistatin-related protein 1* (*fstl1*); *myoblast determination protein 2* (*MyoD2*); *nuclear receptor coactivator 4* (*ncoa4*); *growth hormone 2* (*gh2*); *fatty acid binding protein 6* (*fabp6*); *titin-like* (*ttn*); *four and a half LIM domains protein 1* (*fhl1*); *ubiquitin specific protease 14* (*ubp14*); *heat shock protein 30* (*hsp30*).
(DOCX)

Table S8 **Expression of reproductive-related contigs in red and white skeletal muscle of resting and swimming trout.** Columns represent contig number, contig annotation, sequence length of the contig, read-per-kilo-base-of-exon-model (RPKM) value for swimmers, RPKM value for resters, fold change (fc) expression by RPKM. Abbreviations: *estrogen receptor beta* (*erb*); *follicle-stimulating hormone beta* (*fshb*); *vitellogenin* (*vtg*); *kiss-1 receptor* (*kiss-1r*).
(DOCX)
