# Supplementary figures and images for: Deep RNA Sequencing of the Skeletal Muscle Transcriptome in Swimming Fish
Source: PLoS One. 2013 Jan 8;8(1):e53171. doi: 10.1371/journal.pone.0053171 (PMC3540090; doi:10.1371/journal.pone.0053171)

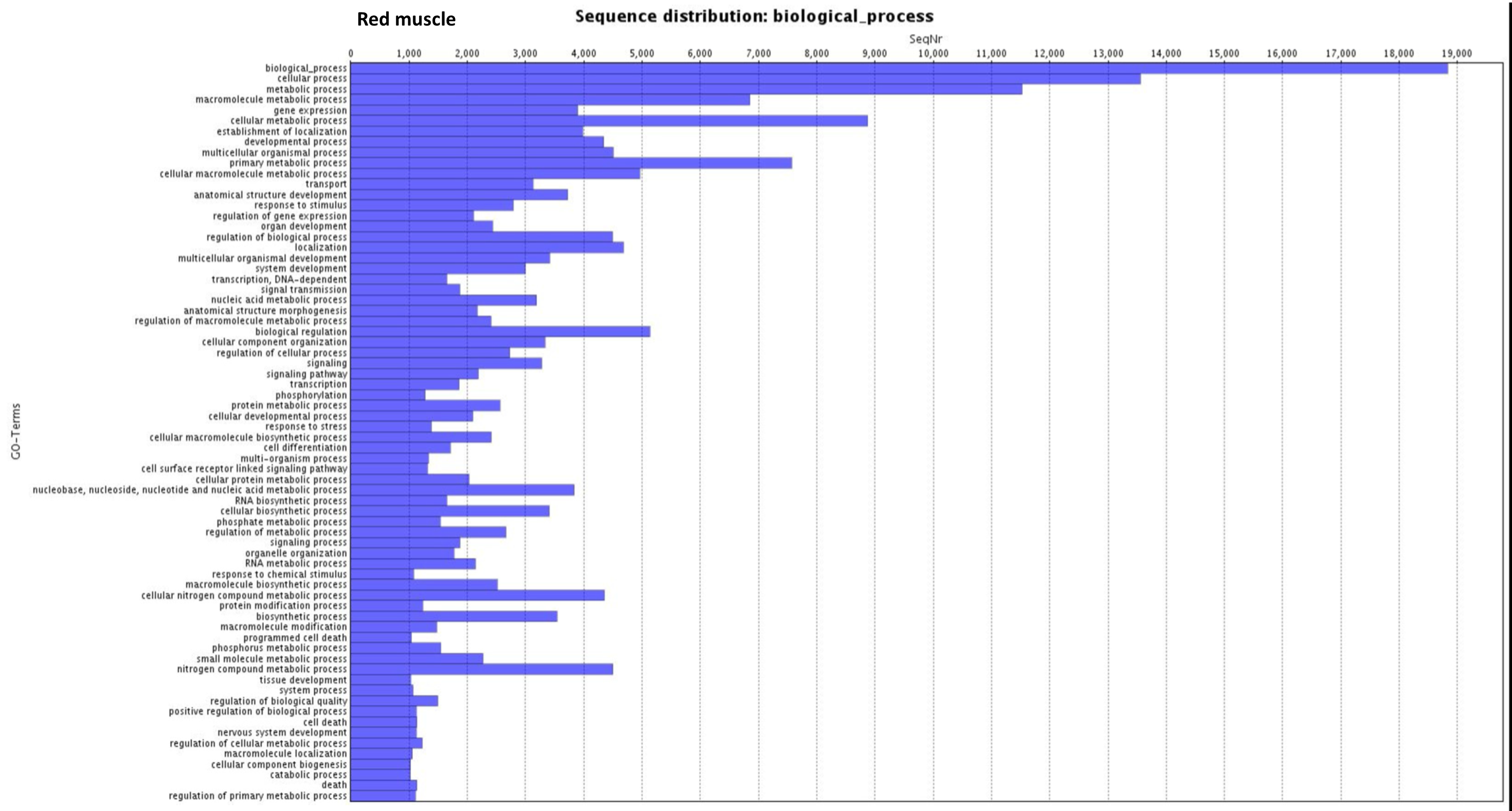

Supplement: Figure S1 — Sequence distribution of GO terms in relation to the number of sequences (SeqNr) of biological processes of the red muscle transcriptome. (TIF) [file pone.0053171.s001.tif]

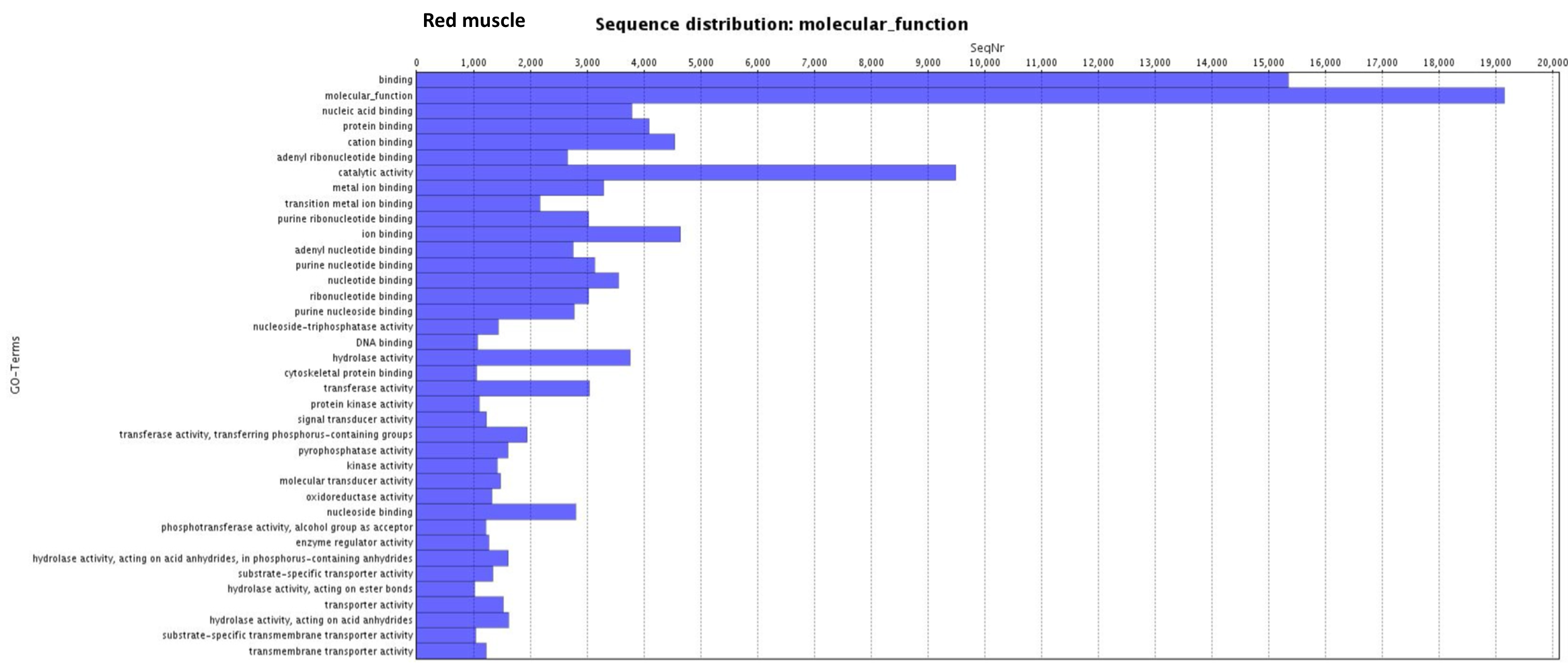

Supplement: Figure S2 — Sequence distribution of GO terms in relation to the number of sequences (SeqNr) of molecular functions of the red muscle transcriptome. (TIF) [file pone.0053171.s002.tif]

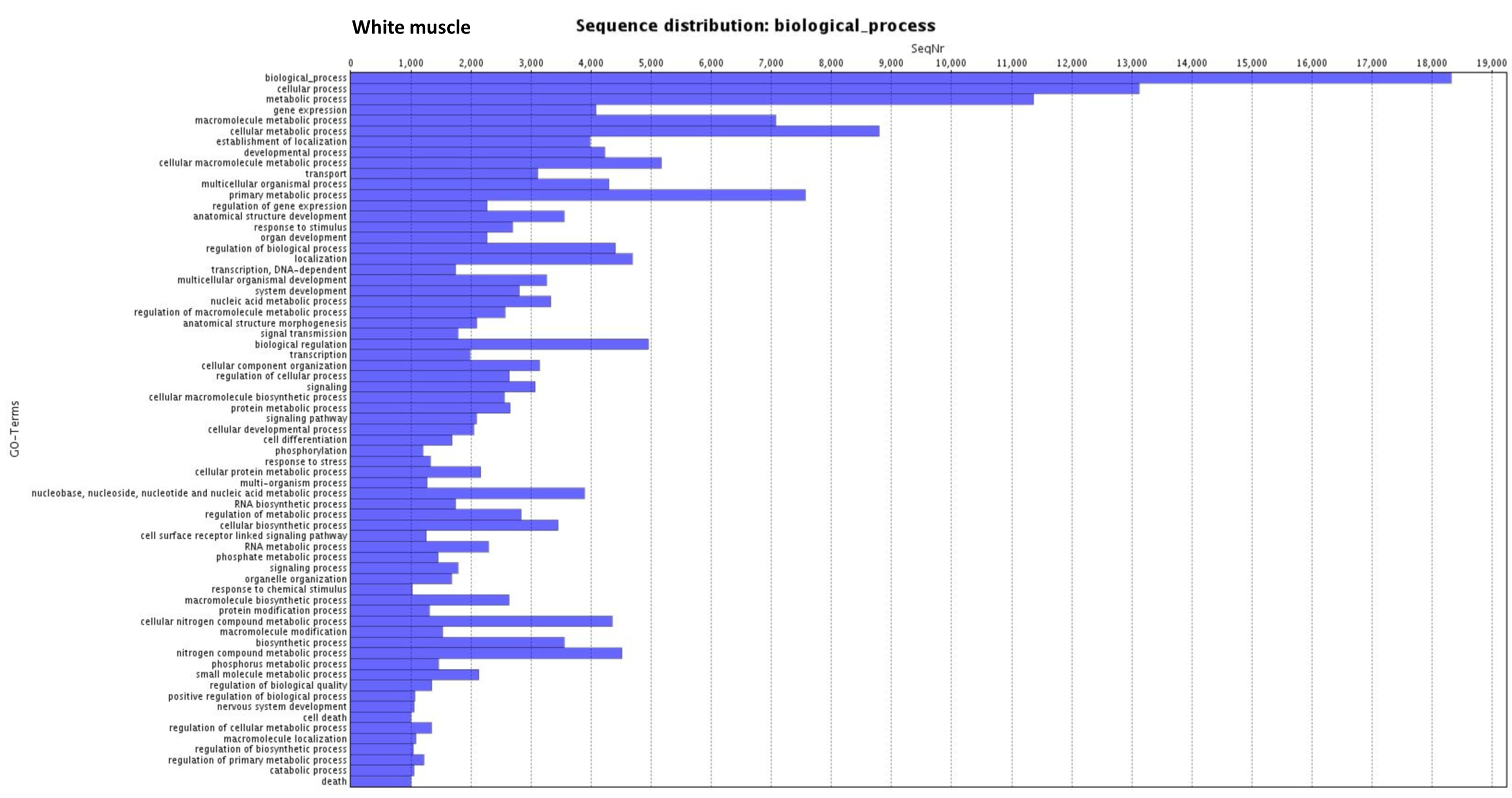

Supplement: Figure S3 — Sequence distribution of GO terms in relation to the number of sequences (SeqNr) of biological processes of the white muscle transcriptome. (TIF) [file pone.0053171.s003.tif]

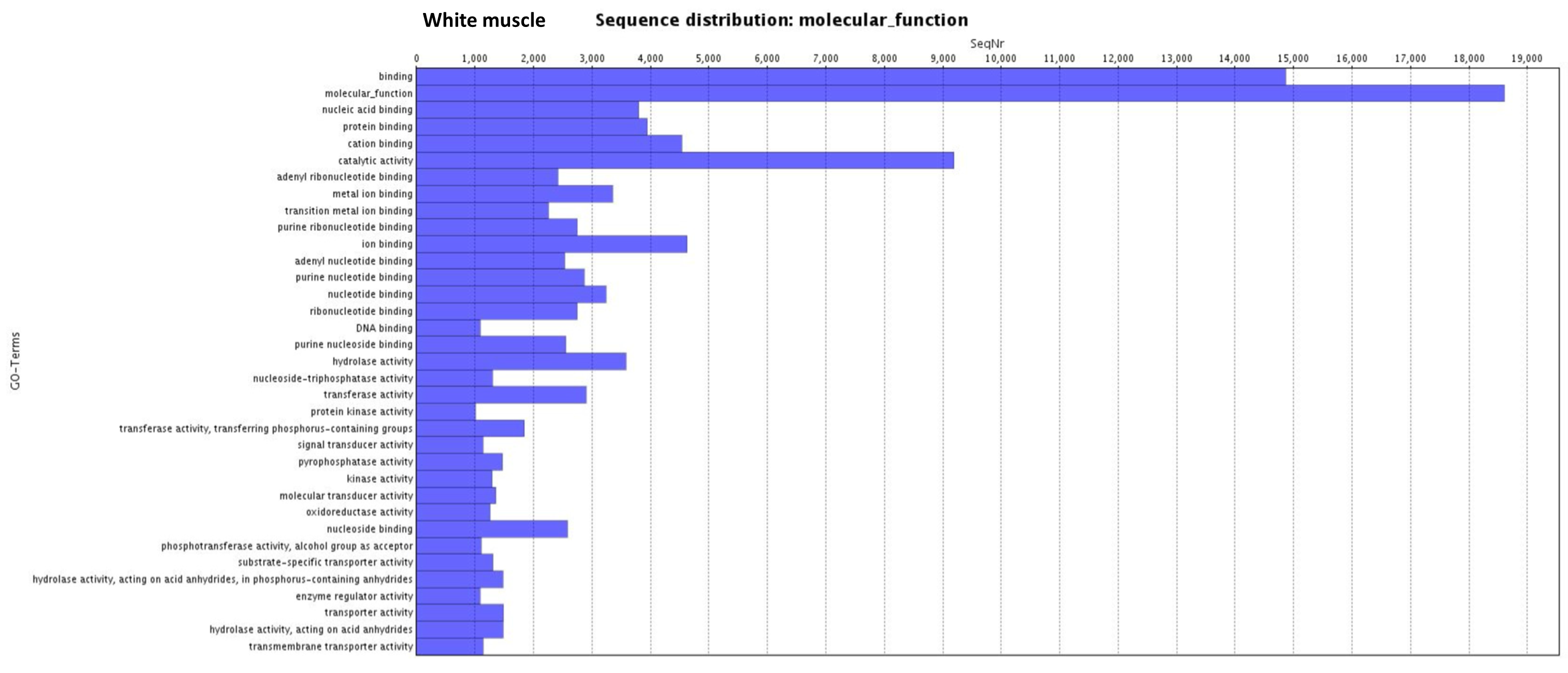

Supplement: Figure S4 — Sequence distribution of GO terms in relation to the number of sequences (SeqNr) of molecular functions of the white muscle transcriptome. (TIF) [file pone.0053171.s004.tif]
