# Supplementary material for: Deep RNA Sequencing of the Skeletal Muscle Transcriptome in Swimming Fish
Source: PLoS One. 2013 Jan 8;8(1):e53171. doi: 10.1371/journal.pone.0053171 (PMC3540090; doi:10.1371/journal.pone.0053171)
Supplement: Table S1 — Nucleotide sequence of primers used for validation of selected target genes by Q-PCR. Forward (F) and reverse (R) primers were designed for the housekeeping gene rps18 (Genbank accession number AF308735) and for target genes on basis of the nucleotide sequence of selected contigs that were larger than 500 nt, that had a SIGENAE salmonid annotation and that were differentially expressed at a fc ≤0.5 or fc ≥2. Primers were designed on basis of the overlapping region between the two sequences for red muscle (R) and white muscle (W) and the same primers were used for both tissues (‘R, W’), on basis of contig sequences that were different for both tissues but were associated with the same genes (‘R’ and ‘W’), or on basis of contig sequences that were tissue specific differentially expressed (‘R’ or ‘W’). (DOCX) [file pone.0053171.s005.docx]

**Table S1**.

| **Target gene** |  | **Muscle** |  | **sequence (5´-3´)** |
| --- | --- | --- | --- | --- |
| *18s ribosomal RNA* | *rps18* | R, W | F | CGGAGGTTCGAAGACGATCA |
|  |  |  | R | TCGCTAGTTGGCATCGTTTAT |
| *guanylate-binding protein* | *gbp* | R, W | F | GGCTGCTGAGGAACAAGTCGT |
|  |  |  | R | GGGATGAATTGGAAAGGGAGAC |
| *troponin T3b, skeletal, fast isoform 1* | *T3b1* | R, W | F | CCGATGATGACGCTAAGAAGAAGT |
|  |  |  | R | GCTTGTCCTCGTTCAGATGGTC |
| *Troponin C, skeletal muscle* | *tropC* | R | F | GCCAGAATCCGACAAGAGAGG |
|  |  |  | R | CGGATGATGATGGCGAACTCT |
|  |  | W | F | GAGCCAGTCACAGAGGAGGAATG |
|  |  |  | R | GGAAGGGTCACAAAGGAATGGT |
| *IgM membrane heavy bound form* | *IgM* | R | F | CCACGAGGTAGACAGGGAAGGA |
|  |  |  | R | CCTCACGCCACCGATGTCCAAT |
|  |  | W | F | CCACGAGGTAGACAGGGAAGGA |
|  |  |  | R | CGATGCCATACCACCAACACC |
| *retinoic acid receptor gamma b* | *Rargb* | R | F | CACCTATCCAGTCCCCCTTCA |
|  |  |  | R | CCACTAAAATGAACCACGACAAGAC |
|  |  | W | F | GTGGCTCAACTTACCCCTCTCC |
|  |  |  | R | GGGTGAAGGAGTGTTTGCGAGT |
| *phosphofructokinase muscle b* | *pfkmb* | R | F | CAGGAAACAGCACACCGAGAGT |
|  |  |  | R | AAACACATACATACCGACCCTTGG |
|  |  | W | F | AATGAACCACCACCTCCACTTG |
|  |  |  | R | CCGACCTAATGCCTGCGACT |
| *follistatin-related protein 1* | *fstl1* | R | F | GCCTGGATACTTGTTCGGATTG |
|  |  |  | R | GAGGAGACACAGGCGTTATCACA |
| *myoblast determination protein 2* | *MyoD2* | R | F | TCACCGACCAACTGCCCACA |
|  |  |  | R | GCCATCCTATTCTTCTACCACAGC |
| *nuclear receptor coactivator 4* | *ncoa4* | R | F | GAGGAGGAAGAGAGGGCAGAGA |
|  |  |  | R | CCCAGGTTCTCAATCTCCACAG |
| *growth hormone 2* | *gh2* | R | F | GCACAAAGACCAAGCCAGGA |
|  |  |  | R | GGGTCTATTTGAGTGAGCCGTGAG |
| *fatty acid binding protein 6* | *fabp6* | R | F | CTGCTTCTCCCTCTCCTTTACCTC |
|  |  |  | R | GCTTGTAGTCACGACCCTTCTCA |
| *titin-like* | *ttn* | W | F | GCATCCCTCTGGCAACTCATC |
|  |  |  | R | GGGAAACACCACTCACAGACGG |
| *four and a half LIM domains protein 1* | *fhl1* | W | F | GCTTGGGTCAAATTCAGAACACTG |
|  |  |  | R | GATTCAGGAAGCATGTAACAGCCA |
| *ubiquitin specific protease 14* | *ubp14* | W | F | GTGCCTCCCTTGACCATAACCT |
|  |  |  | R | CCAACTACACCCGACAGCGAAT |
| *heat shock protein 30* | *hsp30* | W | F | GTCGGTGATGCTTCCCTCTGT |
|  |  |  | R | CTCCACATTCAGGCACCAAAGA |
