# Supplementary material for: Deep RNA Sequencing of the Skeletal Muscle Transcriptome in Swimming Fish
Source: PLoS One. 2013 Jan 8;8(1):e53171. doi: 10.1371/journal.pone.0053171 (PMC3540090; doi:10.1371/journal.pone.0053171)
Supplement: Table S3 — Down regulated contigs (>500 nt) in the red muscle of swimmers. Columns show the number of the specific contig, its annotation, the database from which a BLAST hit was obtained (SIGENAE salmonids, Refseq zebrafish, Refseq metazoa), the length of the contig in nucleotides, the Reads Per Kilobase (exon model) per Million mapped reads (RPKM) value of swimmers, the RPKM value of resters and the fold change of swimmers vs. resters. (DOCX) [file pone.0053171.s007.docx]

**Table S3**.

| ***contig*** | ***annotation*** | ***BLAST hit*** | ***length (nt)*** | ***RPKM swimmers*** | ***RPKM resters*** | ***fc by RPKM*** |
| --- | --- | --- | --- | --- | --- | --- |
| 141742 | ---NA--- |  | 561 | 5.60 | 278.25 | 0.02 |
| 37864 | si:ch211-173b8.2 [D. rerio] | Refseq zebrafish | 894 | 10.25 | 298.52 | 0.03 |
| 38584 | Myosin regulatory light chain 2, ventricular/cardiac muscle isoform [Salmo salar] | SIGENAE salmonids | 618 | 49.76 | 744.18 | 0.07 |
| 36636 | troponin T type 1 (skeletal, slow) [X laevis] | Refseq metazoa | 1,067 | 14.84 | 191.90 | 0.08 |
| 112604 | ---NA--- |  | 906 | 18.78 | 200.65 | 0.09 |
| 45249 | ---NA--- |  | 567 | 6.92 | 54.02 | 0.13 |
| 112349 | PREDICTED: osteocrin-like [X (Silurana) tropicalis] | Refseq metazoa | 1,203 | 24.36 | 179.24 | 0.14 |
| 37356 | kinesin light chain 4 [D. rerio] | Refseq zebrafish | 847 | 6.33 | 45.35 | 0.14 |
| 141253 | tumor protein 63 isoform alpha 2 [D. rerio] | Refseq zebrafish | 1,252 | 11.39 | 59.87 | 0.19 |
| 38051 | ---NA--- |  | 1,377 | 9.22 | 47.53 | 0.19 |
| 112504 | ventricular myosin heavy chain [D. rerio] | Refseq zebrafish | 759 | 12.76 | 59.88 | 0.21 |
| 37892 | ---NA--- |  | 774 | 19.10 | 87.60 | 0.22 |
| 141154 | Salmo salar clone CH214-714P22 MHC Class I (Sasa-UBA) gene, Sasa-UBA*0601 allele, partial cds; and proteasome subunit beta type 8 (PSMB8a), proteasome subunit beta type 10 (PSMB10a), PSMB9a-like (PSMB9a-like), proteosome subunit beta type 9 (PSMB9), transporter 2 ATP binding cassette (TAP2a), bromodomain containing 2 (BRD2), collagen Type XI Alpha2 (COL11A2), retinoid X receptor beta (RXRB), solute carrier 39 (zinc transporter) member 7 (SLC39A7), and reverse transcriptase-like protein genes, complete cds | SIGENAE salmonids | 1,065 | 17.45 | 77.81 | 0.22 |
| 40254 | Salmo salar clone ssal-rgf-527-229 BTG2 putative mRNA, complete cds | SIGENAE salmonids | 506 | 13.71 | 60.04 | 0.23 |
| 1362 | ---NA--- |  | 772 | 6.95 | 30.39 | 0.23 |
| 112900 | predicted protein [Nematostella vectensis] | Refseq metazoa | 571 | 9.63 | 39.80 | 0.24 |
| 38066 | four and a half LIM domains 2a [D. rerio] | Refseq zebrafish | 1,179 | 15.09 | 61.91 | 0.24 |
| 141438 | ventricular myosin heavy chain [D. rerio] | SIGENAE salmonids | 731 | 229.84 | 934.93 | 0.25 |
| 38702 | ---NA--- |  | 619 | 7.61 | 30.13 | 0.25 |
| 112525 | tubulin polymerization-promoting protein family member 3 [Salmo salar] | Refseq metazoa | 672 | 8.57 | 33.82 | 0.25 |
| 39051 | ---NA--- |  | 566 | 9.71 | 36.66 | 0.26 |
| 141261 | peroxisomal proliferator-activated receptor beta1A [Salmo salar] | SIGENAE salmonids | 937 | 10.33 | 38.88 | 0.27 |
| 283 | tumor protein 63 isoform alpha 2 [D. rerio] | Refseq zebrafish | 872 | 12.76 | 46.74 | 0.27 |
| 37786 | novel protein [D. rerio] | SIGENAE salmonids | 604 | 6.07 | 22.08 | 0.27 |
| 45473 | Gastrotropin [Salmo salar] | SIGENAE salmonids | 527 | 37.74 | 133.34 | 0.28 |
| 37249 | AXIN1 up-regulated 1 [X (Silurana) tropicalis] | SIGENAE salmonids | 747 | 8.58 | 30.25 | 0.28 |
| 38365 | PREDICTED: uncharacterized protein KIAA0408-like [Callithrix jacchus] | Refseq metazoa | 763 | 8.06 | 27.84 | 0.29 |
| 38000 | Desmin [Osmerus mordax] | SIGENAE salmonids | 1,327 | 32.44 | 112.05 | 0.29 |
| 37453 | Salmo salar clone ssal-rgf-527-229 BTG2 putative mRNA, complete cds | SIGENAE salmonids | 834 | 15.69 | 53.60 | 0.29 |
| 53929 | alpha-actinin-3 [Equus caballus] | Refseq metazoa | 518 | 10.36 | 35.29 | 0.29 |
| 38607 | ---NA--- |  | 574 | 9.57 | 31.63 | 0.30 |
| 39792 | ---NA--- |  | 541 | 14.03 | 45.66 | 0.31 |
| 38637 | Desmin [Osmerus mordax] | SIGENAE salmonids | 560 | 17.99 | 57.12 | 0.32 |
| 39111 | myoblast determination protein 2 [Oncorhynchus mykiss] | SIGENAE salmonids | 598 | 9.63 | 30.36 | 0.32 |
| 38632 | ---NA--- |  | 816 | 9.94 | 30.57 | 0.33 |
| 42084 | microtubule associated monoxygenase, calponin and LIM domain containing 2, isoform CRA_c [Homo sapiens] | SIGENAE salmonids | 610 | 7.72 | 23.69 | 0.33 |
| 41898 | Oncorhynchus mykiss isolate OMS00038 genomic sequence | SIGENAE salmonids | 544 | 11.31 | 34.51 | 0.33 |
| 112423 | reverse transcriptase [Takifugu rubripes] | SIGENAE salmonids | 893 | 12.16 | 36.93 | 0.33 |
| 38493 | ---NA--- |  | 725 | 5.96 | 18.06 | 0.33 |
| 36762 | Tetraodon nigroviridis full-length cDNA | SIGENAE salmonids | 3,142 | 31.07 | 92.84 | 0.33 |
| 39215 | annexin A5a [D. rerio] | Refseq zebrafish | 632 | 6.00 | 17.78 | 0.34 |
| 37695 | PREDICTED: Seizure related 6 homolog (mouse)-like [B. taurus] | Refseq metazoa | 998 | 14.95 | 43.43 | 0.34 |
| 112878 | ---NA--- |  | 735 | 11.57 | 32.60 | 0.36 |
| 38089 | Myozenin-2 [Salmo salar] | SIGENAE salmonids | 520 | 131.60 | 370.49 | 0.36 |
| 141493 | thymosin beta [D. rerio] | Refseq zebrafish | 599 | 133.47 | 368.84 | 0.36 |
| 38232 | ---NA--- |  | 699 | 272.18 | 752.11 | 0.36 |
| 41593 | Sperm acrosome membrane-associated protein 4 [Salmo salar] | SIGENAE salmonids | 527 | 9.93 | 27.42 | 0.36 |
| 38188 | zinc finger protein [Heliothryx barroti] | SIGENAE salmonids | 584 | 14.79 | 40.18 | 0.37 |
| 37373 | ---NA--- |  | 660 | 6.74 | 18.15 | 0.37 |
| 36675 | Salmo salar homeobox protein HoxB9aa (HoxB9aa), homeobox protein HoxB8aa (HoxB8aa), homeobox protein HoxB7aa (HoxB7aa), homeobox protein HoxB6aa (HoxB6aa), homeobox protein HoxB5aa (HoxB5aa), homeobox protein HoxB4aa (HoxB4aa), homeobox protein HoxB3aa (HoxB3aa), homeobox protein HoxB2aa (HoxB2aa), and homeobox protein HoxB1aa (HoxB1aa) genes, complete cds | SIGENAE salmonids | 1,254 | 18.57 | 49.73 | 0.37 |
| 39835 | myosin, light polypeptide 3-1 [Salmo salar] | SIGENAE salmonids | 771 | 40.90 | 109.08 | 0.37 |
| 142492 | ---NA--- |  | 582 | 7.42 | 19.73 | 0.38 |
| 36888 | PREDICTED: similar to KIAA1561 protein [Monodelphis domestica] | Refseq metazoa | 715 | 16.47 | 43.70 | 0.38 |
| 36657 | Salmo salar clone 242N16 formin-binding protein 1 gene, partial sequence and TCR gamma locus region | SIGENAE salmonids | 2,124 | 13.00 | 34.48 | 0.38 |
| 42196 | Jade3 protein [Takifugu rubripes] | SIGENAE salmonids | 557 | 4.70 | 12.42 | 0.38 |
| 114189 | myosin regulatory light chain 2 [Salmo salar] | SIGENAE salmonids | 683 | 67.24 | 177.38 | 0.38 |
| 114554 | myosin heavy chain [Oncorhynchus mykiss] | SIGENAE salmonids | 532 | 257.52 | 678.54 | 0.38 |
| 37100 | Salmo salar clone 242N16 formin-binding protein 1 gene, partial sequence and TCR gamma locus region | SIGENAE salmonids | 675 | 19.39 | 50.68 | 0.38 |
| 112736 | Troponin I, slow skeletal muscle [Anoplopoma fimbria] | SIGENAE salmonids | 1,071 | 596.70 | 1554.38 | 0.38 |
| 142547 | Myosin light polypeptide 4 [Salmo salar] | SIGENAE salmonids | 666 | 13.75 | 35.42 | 0.39 |
| 141435 | phosphoglucomutase 5 [D. rerio] | SIGENAE salmonids | 547 | 46.41 | 118.08 | 0.39 |
| 112344 | PREDICTED: similar to KIAA1561 protein [Monodelphis domestica] | Refseq metazoa | 594 | 11.68 | 29.52 | 0.40 |
| 39566 | ---NA--- |  | 562 | 8.38 | 21.10 | 0.40 |
| 113266 | ---NA--- |  | 605 | 14.71 | 36.95 | 0.40 |
| 113536 | OTU domain-containing protein 6B [D. rerio] | Refseq zebrafish | 687 | 12.57 | 31.46 | 0.40 |
| 115011 | myosin heavy chain [Oncorhynchus keta] | SIGENAE salmonids | 519 | 140.93 | 352.64 | 0.40 |
| 214 | ---NA--- |  | 804 | 17.90 | 44.55 | 0.40 |
| 43250 | formin-binding protein 1 [D. rerio] | Refseq zebrafish | 711 | 17.12 | 42.38 | 0.40 |
| 115702 | ---NA--- |  | 562 | 5.36 | 13.19 | 0.41 |
| 41008 | ---NA--- |  | 573 | 8.68 | 21.34 | 0.41 |
| 36871 | envelope protein [Atlantic salmon swim bladder sarcoma virus] | SIGENAE salmonids | 1,490 | 23.54 | 57.52 | 0.41 |
| 37033 | novel protein [D. rerio] | SIGENAE salmonids | 716 | 14.07 | 34.32 | 0.41 |
| 114142 | troponin T3b, skeletal, fast isoform 1 [D. rerio] | SIGENAE salmonids | 762 | 15.80 | 38.25 | 0.41 |
| 45920 | Follistatin-related protein 1 [Salmo salar] | SIGENAE salmonids | 569 | 6.67 | 16.06 | 0.42 |
| 43423 | ---NA--- |  | 558 | 26.50 | 63.74 | 0.42 |
| 84660 | Diamine acetyltransferase 1 [Salmo salar] | SIGENAE salmonids | 621 | 10.96 | 26.25 | 0.42 |
| 141211 | PREDICTED: uveal autoantigen with coiled-coil domains and ankyrin repeats isoform 1 [Macaca mulatta] | Refseq metazoa | 1,149 | 19.36 | 46.22 | 0.42 |
| 38388 | ---NA--- |  | 778 | 49.28 | 117.15 | 0.42 |
| 36937 | myosin, light polypeptide 3-3 [Salmo salar] | SIGENAE salmonids | 977 | 57.86 | 136.52 | 0.42 |
| 41474 | ---NA--- |  | 662 | 12.06 | 28.36 | 0.43 |
| 36739 | TMyogenin protein [Oncorhynchus mykiss] | SIGENAE salmonids | 1,581 | 16.88 | 38.74 | 0.44 |
| 39020 | Heme oxygenase [Salmo salar] | SIGENAE salmonids | 1,121 | 20.19 | 46.16 | 0.44 |
| 112323 | Mitogen-activated protein kinase kinase kinase 14 [Salmo salar] | SIGENAE salmonids | 3,597 | 99.09 | 224.51 | 0.44 |
| 112959 | Cellular retinoic acid-binding protein [Salmo salar] | SIGENAE salmonids | 612 | 31.86 | 72.04 | 0.44 |
| 141437 | troponin T2a, cardiac [D. rerio] | Refseq zebrafish | 891 | 241.14 | 543.47 | 0.44 |
| 38643 | Zebrafish DNA sequence from clone CH211-65B3, complete sequence | SIGENAE salmonids | 624 | 41.94 | 94.21 | 0.45 |
| 113802 | PREDICTED: similar to ORF2-encoded protein [Strongylocentrotus purpuratus] | Refseq metazoa | 502 | 8.60 | 19.19 | 0.45 |
| 37209 | PREDICTED: similar to sallimus CG1915-PC, isoform C [Apis mellifera] | Refseq metazoa | 971 | 15.36 | 34.21 | 0.45 |
| 141279 | heat shock 27kDa protein 1 transcript variant 2 [Oncorhynchus mykiss] | SIGENAE salmonids | 1,258 | 66.26 | 146.96 | 0.45 |
| 36859 | ---NA--- |  | 1,050 | 25.30 | 56.10 | 0.45 |
| 113286 | Mitogen-activated protein kinase kinase kinase 14 [Salmo salar] | SIGENAE salmonids | 640 | 76.87 | 169.62 | 0.45 |
| 44953 | novel slow skeletal troponin T family protein [D. rerio] | SIGENAE salmonids | 1,006 | 383.05 | 841.52 | 0.46 |
| 113279 | ---NA--- |  | 594 | 10.79 | 23.70 | 0.46 |
| 36750 | Troponin C, skeletal muscle [Salmo salar] | SIGENAE salmonids | 932 | 48.44 | 105.74 | 0.46 |
| 113109 | ---NA--- |  | 514 | 10.69 | 23.31 | 0.46 |
| 41715 | ---NA--- |  | 775 | 9.46 | 20.56 | 0.46 |
| 113322 | ---NA--- |  | 997 | 32.15 | 69.86 | 0.46 |
| 36773 | ---NA--- |  | 1,778 | 19.65 | 42.23 | 0.47 |
| 45924 | fibroblast growth factor 1 (acidic) [D. rerio] | Refseq zebrafish | 659 | 28.99 | 61.84 | 0.47 |
| 37284 | Myozenin-2 [Salmo salar] | SIGENAE salmonids | 962 | 8.98 | 19.13 | 0.47 |
| 115959 | ---NA--- |  | 540 | 10.42 | 22.18 | 0.47 |
| 38951 | Salmo salar retinoic acid receptor gamma b (Rargb) gene, partial cds; and homeobox protein HoxC13ba (HoxC13ba), homeobox protein HoxC12ba (HoxC12ba), homeobox protein HoxC11ba (HoxC11ba), homeobox protein HoxC10ba (HoxC10ba), homeobox protein HoxC9ba (HoxC9ba), homeobox protein HoxC8ba (HoxC8ba), homeobox protein HoxC6ba (HoxC6ba), homeobox protein HoxC5ba (HoxC5ba), and homeobox protein HoxC4ba (HoxC4ba) genes, complete cds | SIGENAE salmonids | 928 | 23.41 | 49.77 | 0.47 |
| 78484 | ---NA--- |  | 605 | 16.44 | 34.91 | 0.47 |
| 142978 | PREDICTED: laminin subunit beta-2-like [X (Silurana) tropicalis] | Refseq metazoa | 541 | 9.68 | 20.55 | 0.47 |
| 3031 | Salmo salar Follistatin-related protein 1 (fstl1), mRNA | SIGENAE salmonids | 806 | 17.05 | 36.16 | 0.47 |
| 37088 | ---NA--- |  | 1,232 | 29.63 | 62.65 | 0.47 |
| 112521 | RIKEN cDNA 5730593F17 [Mus musculus] | SIGENAE salmonids | 645 | 23.13 | 48.44 | 0.48 |
| 84450 | Transcription factor AP-1 [Salmo salar] | SIGENAE salmonids | 684 | 13.77 | 28.71 | 0.48 |
| 47588 | ---NA--- |  | 556 | 15.06 | 31.32 | 0.48 |
| 113351 | PREDICTED: similar to dentin matrix acidic phosphoprotein isoform 2 [Equus caballus] | Refseq metazoa | 642 | 37.71 | 77.91 | 0.48 |
| 112617 | PREDICTED: myomegalin-like [Ailuropoda melanoleuca] | Refseq metazoa | 901 | 47.34 | 97.45 | 0.49 |
| 113081 | ---NA--- |  | 513 | 8.93 | 18.30 | 0.49 |
| 39179 | alpha-actinin-2 [D. rerio] | Refseq zebrafish | 944 | 33.96 | 69.60 | 0.49 |
| 114130 | ---NA--- |  | 725 | 27.79 | 56.72 | 0.49 |
| 141803 | rCG35999, isoform CRA_b [Rattus norvegicus] | SIGENAE salmonids | 674 | 60.57 | 122.58 | 0.49 |
| 141270 | Tetraodon nigroviridis full-length cDNA | SIGENAE salmonids | 570 | 38.11 | 76.92 | 0.50 |
| 141233 | ---NA--- |  | 774 | 26.20 | 52.81 | 0.50 |
| 141182 | kelch repeat and BTB (POZ) domain containing 10 [D. rerio] | SIGENAE salmonids | 1,949 | 419.40 | 841.22 | 0.50 |
