# Supplementary material for: Deep RNA Sequencing of the Skeletal Muscle Transcriptome in Swimming Fish
Source: PLoS One. 2013 Jan 8;8(1):e53171. doi: 10.1371/journal.pone.0053171 (PMC3540090; doi:10.1371/journal.pone.0053171)
Supplement: Table S4 — Up regulated contigs (>500 nt) in the red muscle of swimmers. See legend Table S3 for a description. (DOCX) [file pone.0053171.s008.docx]

**Table S4**.

| ***contig*** | ***annotation*** | ***BLAST hit*** | ***length (nt)*** | ***RPKM swimmers*** | ***RPKM resters*** | ***fc by RPKM*** |
| --- | --- | --- | --- | --- | --- | --- |
| 44289 | Abhydrolase domain-containing protein 6 [Salmo salar] | Refseq metazoa | 508 | 21.38 | 1.95 | 10.99 |
| 142086 | guanylate-binding protein [Oncorhynchus mykiss] | SIGENAE salmonids | 533 | 84.70 | 8.81 | 9.62 |
| 141358 | PREDICTED: caseinolytic peptidase B [Oryctolagus cuniculus] | Refseq metazoa | 623 | 28.77 | 4.16 | 6.91 |
| 9370 | guanylate-binding protein [Oncorhynchus mykiss] | SIGENAE salmonids | 558 | 30.25 | 4.43 | 6.83 |
| 37437 | IgM heavy chain membrane bound form [Oncorhynchus mykiss] | SIGENAE salmonids | 744 | 56.28 | 11.29 | 4.99 |
| 39250 | predicted protein [Nematostella vectensis] | Refseq metazoa | 652 | 19.47 | 4.74 | 4.11 |
| 112524 | ---NA--- |  | 1,060 | 66.66 | 21.55 | 3.09 |
| 41489 | Cytoplasmic FMR1-interacting protein 1 homolog [Salmo salar] | SIGENAE salmonids | 618 | 17.79 | 5.80 | 3.07 |
| 38673 | ---NA--- |  | 620 | 62.05 | 21.11 | 2.94 |
| 141293 | ---NA--- |  | 1,402 | 210.09 | 73.29 | 2.87 |
| 83937 | myomesin 1, 185kDa [Gallus gallus] | SIGENAE salmonids | 666 | 31.04 | 11.50 | 2.70 |
| 41793 | RAS-like family 11 member B [D. rerio] | SIGENAE salmonids | 615 | 20.00 | 7.63 | 2.62 |
| 114289 | ---NA--- |  | 662 | 34.20 | 13.06 | 2.62 |
| 36716 | LIM and cysteine-rich domains protein 1 [B. taurus] | SIGENAE salmonids | 1,390 | 62.04 | 24.61 | 2.52 |
| 141737 | Lysozyme g [Oncorhynchus mykiss] | SIGENAE salmonids | 760 | 29.79 | 12.03 | 2.48 |
| 37836 | ---NA--- |  | 554 | 18.66 | 7.58 | 2.46 |
| 47204 | TSC22 domain family protein 3 [Salmo salar] | SIGENAE salmonids | 619 | 29.59 | 12.17 | 2.43 |
| 85972 | RIKEN cDNA 2610528E23 [Mus musculus] | SIGENAE salmonids | 997 | 37.14 | 15.36 | 2.42 |
| 39790 | proteasome subunit [Oncorhynchus mykiss] | SIGENAE salmonids | 574 | 20.97 | 8.82 | 2.38 |
| 40962 | Salmo salar clone 63I10 growth hormone 2 gene, complete cds; and skeletal muscle sodium channel alpha subunit-like, myosin alkali light chain-like, and microtubule-associated protein Tau-like genes, complete sequence | SIGENAE salmonids | 579 | 19.21 | 8.11 | 2.37 |
| 9627 | PREDICTED: zinc finger protein 638, partial [Taeniopygia guttata] | Refseq metazoa | 829 | 32.83 | 13.85 | 2.37 |
| 43277 | D. rerio adenosylmethionine decarboxylase 1 (amd1), mRNA | SIGENAE salmonids | 515 | 22.61 | 9.59 | 2.36 |
| 42067 | insulin-like growth factor binding protein 5a [D. rerio] | Refseq zebrafish | 565 | 21.08 | 9.18 | 2.30 |
| 84393 | MF-25 protein [Schistosoma japonicum] | SIGENAE salmonids | 530 | 54.32 | 24.00 | 2.26 |
| 113786 | PREDICTED: kelch-like 22 (Drosophila) [Taeniopygia guttata] | Refseq metazoa | 681 | 15.56 | 6.89 | 2.26 |
| 115089 | 2-acylglycerol O-acyltransferase 2 [D. rerio] | Refseq zebrafish | 651 | 42.21 | 18.78 | 2.25 |
| 142218 | ---NA--- |  | 510 | 13.60 | 6.05 | 2.25 |
| 112775 | matrin 3 [X (Silurana) tropicalis] | SIGENAE salmonids | 598 | 273.95 | 122.05 | 2.24 |
| 39261 | ---NA--- |  | 844 | 26.51 | 11.85 | 2.24 |
| 39398 | ---NA--- |  | 696 | 18.61 | 8.34 | 2.23 |
| 85969 | PREDICTED: similar to BCR variant protein [Taeniopygia guttata] | Refseq metazoa | 945 | 36.28 | 16.47 | 2.20 |
| 38540 | Cytoplasmic FMR1-interacting protein 1 homolog [Salmo salar] | SIGENAE salmonids | 630 | 18.49 | 8.43 | 2.19 |
| 39820 | aconitate hydratase, mitochondrial [D. rerio] | Refseq zebrafish | 507 | 80.26 | 36.78 | 2.18 |
| 40308 | phosphofructokinase, muscle b [D. rerio] | SIGENAE salmonids | 591 | 21.70 | 10.03 | 2.16 |
| 37124 | PREDICTED: NUAK family, SNF1-like kinase, 2 [Taeniopygia guttata] | Refseq metazoa | 1,348 | 31.35 | 14.57 | 2.15 |
| 37322 | Jph2 protein [D. rerio] | SIGENAE salmonids | 589 | 38.66 | 18.03 | 2.14 |
| 41724 | ---NA--- |  | 508 | 23.70 | 11.18 | 2.12 |
| 43429 | MHC class I heavy chain precursor [Oncorhynchus mykiss] | SIGENAE salmonids | 826 | 55.44 | 26.17 | 2.12 |
| 112644 | ---NA--- |  | 656 | 31.32 | 14.87 | 2.11 |
| 38307 | aconitate hydratase, mitochondrial [D. rerio] | Refseq zebrafish | 647 | 91.82 | 43.71 | 2.10 |
| 39129 | ---NA--- |  | 501 | 48.32 | 23.42 | 2.06 |
| 39140 | zinc finger protein 638 isoform 2 [Mus musculus] | Refseq metazoa | 1,005 | 83.85 | 40.67 | 2.06 |
| 40144 | Nuclear receptor coactivator 4 [Salmo salar] | SIGENAE salmonids | 643 | 24.42 | 11.91 | 2.05 |
| 142203 | Protein-glutamine gamma-glutamyltransferase 2 [Salmo salar] | SIGENAE salmonids | 659 | 16.08 | 7.87 | 2.04 |
| 42895 | CD80-like protein [Oncorhynchus mykiss] | SIGENAE salmonids | 520 | 13.59 | 6.65 | 2.04 |
| 141456 | myosin-7B [Gallus gallus] | Refseq metazoa | 1,012 | 116.50 | 57.36 | 2.03 |
| 38179 | ---NA--- |  | 658 | 71.99 | 35.47 | 2.03 |
| 112770 | chaperone activity of bc1 complex-like, mitochondrial [D. rerio] | SIGENAE salmonids | 1,514 | 214.68 | 106.04 | 2.02 |
| 42448 | zinc finger protein ZFP161 [Homo sapiens] | SIGENAE salmonids | 591 | 13.06 | 6.48 | 2.02 |
| 38204 | PREDICTED: similar to adiponectin, C1Q and collagen domain containing [Equus caballus] | Refseq metazoa | 643 | 35.61 | 17.67 | 2.02 |
| 37984 | Zebrafish DNA sequence from clone CH73-46J18 in linkage group 10, complete sequence | SIGENAE salmonids | 956 | 28.74 | 14.34 | 2.00 |
