# Supplementary material for: Deep RNA Sequencing of the Skeletal Muscle Transcriptome in Swimming Fish
Source: PLoS One. 2013 Jan 8;8(1):e53171. doi: 10.1371/journal.pone.0053171 (PMC3540090; doi:10.1371/journal.pone.0053171)
Supplement: Table S5 — Down regulated contigs (>500 nt) in the white muscle of swimmers. See legend Table S3 for a description. (DOCX) [file pone.0053171.s009.docx]

**Table S5**.

| ***contig*** | ***annotation*** | ***BLAST hit*** | ***length (nt)*** | ***RPKM swimmers*** | ***RPKM resters*** | ***fc by RPKM*** |
| --- | --- | --- | --- | --- | --- | --- |
| 64953 | RecName: Full=Heat shock protein 30; Short=HSP 30 | SIGENAE salmonids | 820 | 7.67 | 296.96 | 0.03 |
| 29635 | ankyrin repeat domain 1 [Salmo salar] | SIGENAE salmonids | 1,619 | 2.56 | 75.75 | 0.03 |
| 65693 | ankyrin repeat domain 1 [Salmo salar] | SIGENAE salmonids | 562 | 1.27 | 32.72 | 0.04 |
| 29589 | RecName: Full=Vitellogenin; Short=VTG; Contains: RecName: Full=Lipovitellin I; Short=LVI; Contains: RecName: Full=Phosvitin; Short=PV; Contains: RecName: Full=Lipovitellin II; Short=LVII; Flags: Precursor | SIGENAE salmonids | 742 | 4.63 | 91.54 | 0.05 |
| 88302 | rRNA promoter binding protein [Rattus norvegicus] | SIGENAE salmonids | 694 | 517.25 | 6138.35 | 0.08 |
| 64907 | heat shock protein 70 [Oryzias latipes] | Refseq metazoa | 2,418 | 32.53 | 160.75 | 0.20 |
| 112938 | Zebrafish DNA sequence from clone DKEY-85K7 in linkage group 7, complete sequence | SIGENAE salmonids | 553 | 6.47 | 28.69 | 0.23 |
| 7689 | rRNA promoter binding protein [Rattus norvegicus] | SIGENAE salmonids | 500 | 14.87 | 65.25 | 0.23 |
| 112007 | titin isoform novex-2 [Homo sapiens] | Refseq metazoa | 646 | 5.54 | 23.19 | 0.24 |
| 1997 | rRNA promoter binding protein [Rattus norvegicus] | SIGENAE salmonids | 522 | 13.43 | 55.12 | 0.24 |
| 112687 | PREDICTED: similar to titin isoform N2-A [Gallus gallus] | Refseq metazoa | 518 | 6.63 | 25.19 | 0.26 |
| 33049 | PREDICTED: similar to titin isoform novex-1 [Gallus gallus] | Refseq metazoa | 732 | 5.86 | 21.68 | 0.27 |
| 29793 | PREDICTED: similar to Titin (Connectin) (Rhabdomyosarcoma antigen MU-RMS-40.14) [Gallus gallus] | Refseq metazoa | 808 | 9.56 | 33.22 | 0.29 |
| 111827 | phosphofructokinase, muscle b [D. rerio] | SIGENAE salmonids | 1,985 | 39.27 | 122.67 | 0.32 |
| 43087 | PREDICTED: titin [B. taurus] | Refseq metazoa | 941 | 9.27 | 28.68 | 0.32 |
| 66394 | Transmembrane protein 9B precursor [Salmo salar] | SIGENAE salmonids | 580 | 1011.22 | 3038.58 | 0.33 |
| 29587 | PREDICTED: titin [B. taurus] | Refseq metazoa | 1,057 | 8.66 | 25.54 | 0.34 |
| 34908 | PREDICTED: titin [B. taurus] | Refseq metazoa | 588 | 6.32 | 18.41 | 0.34 |
| 28941 | PREDICTED: similar to Titin (Connectin) (Rhabdomyosarcoma antigen MU-RMS-40.14) [Monodelphis domestica] | Refseq metazoa | 1,226 | 8.75 | 25.28 | 0.35 |
| 30689 | adipose differentiation-related protein [Anas platyrhynchos] | SIGENAE salmonids | 706 | 6.48 | 18.49 | 0.35 |
| 47890 | PREDICTED: similar to titin [Canis familiaris] | Refseq metazoa | 605 | 6.15 | 17.40 | 0.35 |
| 33663 | ---NA--- |  | 536 | 9.61 | 26.28 | 0.37 |
| 88861 | PREDICTED: similar to titin isoform N2-A [Gallus gallus] | Refseq metazoa | 729 | 8.24 | 22.38 | 0.37 |
| 96091 | Salmo salar clone 31E09 TCR-alpha/delta locus, genomic sequence | SIGENAE salmonids | 593 | 723.76 | 1886.36 | 0.38 |
| 29814 | PREDICTED: titin [B. taurus] | Refseq metazoa | 652 | 10.31 | 26.84 | 0.38 |
| 34205 | PREDICTED: similar to titin [Equus caballus] | Refseq metazoa | 651 | 13.18 | 33.94 | 0.39 |
| 112491 | collagen alpha-2(I) chain precursor [Oncorhynchus mykiss] | SIGENAE salmonids | 744 | 23.64 | 60.20 | 0.39 |
| 28907 | PREDICTED: similar to Titin (Connectin) (Rhabdomyosarcoma antigen MU-RMS-40.14) [Gallus gallus] | Refseq metazoa | 3,257 | 13.52 | 34.42 | 0.39 |
| 112487 | PREDICTED: similar to titin isoform N2-A [Gallus gallus] | Refseq metazoa | 605 | 9.22 | 22.80 | 0.40 |
| 30720 | PREDICTED: similar to Titin (Connectin) (Rhabdomyosarcoma antigen MU-RMS-40.14) [Ornithorhynchus anatinus] | Refseq metazoa | 509 | 8.71 | 21.27 | 0.41 |
| 40698 | PREDICTED: similar to titin isoform novex-1 [Gallus gallus] | Refseq metazoa | 549 | 11.20 | 27.01 | 0.41 |
| 112523 | collagen alpha-2(I) chain precursor [Oncorhynchus mykiss] | SIGENAE salmonids | 656 | 22.67 | 54.26 | 0.42 |
| 97389 | Apoptosis-related protein 3 [Salmo salar] | SIGENAE salmonids | 643 | 22.24 | 52.82 | 0.42 |
| 33951 | titin isoform N2-A [Mus musculus] | Refseq metazoa | 594 | 23.60 | 55.67 | 0.42 |
| 33848 | PREDICTED: similar to titin [Ornithorhynchus anatinus] | Refseq metazoa | 632 | 8.83 | 20.65 | 0.43 |
| 30103 | PREDICTED: similar to titin isoform N2-A [Gallus gallus] | Refseq metazoa | 628 | 14.58 | 33.77 | 0.43 |
| 112145 | 85 kDa calcium-independent phospholipase A2 [D. rerio] | Refseq zebrafish | 504 | 10.22 | 23.54 | 0.43 |
| 34616 | PREDICTED: similar to titin [Ornithorhynchus anatinus] | Refseq metazoa | 501 | 10.28 | 23.38 | 0.44 |
| 34759 | PREDICTED: titin [Rattus norvegicus] | Refseq metazoa | 520 | 11.83 | 26.81 | 0.44 |
| 32648 | PREDICTED: titin [Rattus norvegicus] | Refseq metazoa | 612 | 9.35 | 21.08 | 0.44 |
| 112689 | ---NA--- |  | 734 | 8.96 | 20.20 | 0.44 |
| 30085 | PREDICTED: similar to titin isoform novex-1 [Gallus gallus] | Refseq metazoa | 536 | 9.34 | 21.03 | 0.44 |
| 11091 | PREDICTED: similar to titin isoform N2-A [Gallus gallus] | Refseq metazoa | 546 | 17.29 | 38.57 | 0.45 |
| 112992 | sorbin and SH3 domain-containing protein 1 isoform 4 [Homo sapiens] | Refseq metazoa | 561 | 13.26 | 29.34 | 0.45 |
| 31318 | alpha 1 type I collagen [Oncorhynchus mykiss] | SIGENAE salmonids | 520 | 33.83 | 74.72 | 0.45 |
| 37733 | PREDICTED: similar to titin isoform novex-1 [Gallus gallus] | Refseq metazoa | 627 | 13.00 | 28.38 | 0.46 |
| 29786 | protein-arginine deiminase type-2 [D. rerio] | Refseq zebrafish | 1,044 | 14.38 | 31.39 | 0.46 |
| 88679 | PREDICTED: xin actin-binding repeat-containing protein 2-like [X (Silurana) tropicalis] | Refseq metazoa | 511 | 16.23 | 35.41 | 0.46 |
| 87911 | Immediate early response gene 2 protein [Salmo salar] | SIGENAE salmonids | 793 | 16.95 | 36.84 | 0.46 |
| 29098 | 26S proteasome non-ATPase regulatory subunit 11 [Salmo salar] | SIGENAE salmonids | 566 | 30.57 | 65.77 | 0.46 |
| 36579 | thymosin beta [D. rerio] | Refseq zebrafish | 574 | 31.39 | 67.17 | 0.47 |
| 65017 | collagen a3(I) [Oncorhynchus mykiss] | SIGENAE salmonids | 677 | 34.22 | 73.16 | 0.47 |
| 112011 | novel protein similar to H.sapiens TTN, titin (TTN) [D. rerio] | SIGENAE salmonids | 577 | 17.10 | 36.50 | 0.47 |
| 33409 | PREDICTED: similar to Titin (Connectin) (Rhabdomyosarcoma antigen MU-RMS-40.14) [Ornithorhynchus anatinus] | Refseq metazoa | 513 | 14.50 | 30.93 | 0.47 |
| 111731 | xin actin-binding repeat containing 1 [D. rerio] | SIGENAE salmonids | 7,234 | 71.43 | 151.60 | 0.47 |
| 5672 | PREDICTED: similar to titin [Equus caballus] | Refseq metazoa | 540 | 12.98 | 27.46 | 0.47 |
| 29521 | PREDICTED: similar to Titin (Connectin) (Rhabdomyosarcoma antigen MU-RMS-40.14) [Ornithorhynchus anatinus] | Refseq metazoa | 1,238 | 16.52 | 34.86 | 0.47 |
| 87722 | PREDICTED: similar to Titin (Connectin) (Rhabdomyosarcoma antigen MU-RMS-40.14) [Gallus gallus] | Refseq metazoa | 755 | 16.67 | 35.16 | 0.47 |
| 29584 | PREDICTED: similar to titin isoform N2-A [Gallus gallus] | Refseq metazoa | 1,024 | 22.21 | 46.63 | 0.48 |
| 33532 | titin isoform N2-A [Mus musculus] | Refseq metazoa | 806 | 8.87 | 18.58 | 0.48 |
| 30465 | ubiquitin specific protease 14 [D. rerio] | SIGENAE salmonids | 739 | 31.16 | 64.82 | 0.48 |
| 33596 | BCL2-like 13 (apoptosis facilitator) [D. rerio] | SIGENAE salmonids | 647 | 22.99 | 47.68 | 0.48 |
| 114166 | C-Myc-binding protein [Oncorhynchus mykiss] | SIGENAE salmonids | 565 | 434.87 | 900.55 | 0.48 |
| 111882 | PREDICTED: similar to titin isoform N2-A [Gallus gallus] | Refseq metazoa | 2,352 | 16.24 | 33.42 | 0.49 |
| 33011 | formin homology 2 domain containing 1 [X laevis] | Refseq metazoa | 840 | 10.39 | 21.36 | 0.49 |
| 33810 | titin isoform N2-A [Mus musculus] | Refseq metazoa | 2,264 | 19.65 | 40.22 | 0.49 |
| 29086 | titin-like [D. rerio] | SIGENAE salmonids | 2,886 | 21.41 | 43.68 | 0.49 |
| 29565 | titin-like [D. rerio] | SIGENAE salmonids | 1,040 | 16.50 | 33.37 | 0.49 |
| 113265 | Heat shock protein beta-11 [Salmo salar] | SIGENAE salmonids | 520 | 962.61 | 1942.14 | 0.50 |
| 112650 | PREDICTED: similar to titin isoform N2-A [Gallus gallus] | Refseq metazoa | 507 | 18.34 | 36.86 | 0.50 |
| 115233 | si:dkey-151c10.1 [D. rerio] | Refseq zebrafish | 590 | 17.45 | 34.94 | 0.50 |
