# Supplementary material for: Deep RNA Sequencing of the Skeletal Muscle Transcriptome in Swimming Fish
Source: PLoS One. 2013 Jan 8;8(1):e53171. doi: 10.1371/journal.pone.0053171 (PMC3540090; doi:10.1371/journal.pone.0053171)
Supplement: Table S6 — Up regulated contigs (>500 nt) in the white muscle of swimmers. See legend Table S3 for a description. (DOCX) [file pone.0053171.s010.docx]

**Table S6**.

| ***contig*** | ***annotation*** | ***BLAST hit*** | ***length (nt)*** | ***RPKM swimmers*** | ***RPKM resters*** | ***fc by RPKM*** |
| --- | --- | --- | --- | --- | --- | --- |
| 28989 | IgM heavy chain membrane bound form [Oncorhynchus mykiss] | SIGENAE salmonids | 652 | 95.42 | 19.11 | 4.99 |
| 30629 | ---NA--- |  | 575 | 22.39 | 5.93 | 3.77 |
| 42389 | Si:ch211-197g15.7 protein [D. rerio] | SIGENAE salmonids | 551 | 23.88 | 7.27 | 3.29 |
| 30004 | Four and a half LIM domains protein 1 [Osmerus mordax] | SIGENAE salmonids | 568 | 39.78 | 14.62 | 2.72 |
| 4604 | cationic trypsin-3 [D. rerio] | Refseq zebrafish | 509 | 31.19 | 11.65 | 2.68 |
| 87861 | Troponin C, skeletal muscle [Salmo salar] | SIGENAE salmonids | 605 | 98.10 | 37.75 | 2.60 |
| 29510 | collagen alpha-1(XIX) chain precursor [Homo sapiens] | Refseq metazoa | 527 | 21.17 | 8.44 | 2.51 |
| 87793 | ---NA--- |  | 505 | 24.92 | 9.98 | 2.50 |
| 113149 | troponin T3b, skeletal, fast isoform 1 [D. rerio] | SIGENAE salmonids | 538 | 459.62 | 190.47 | 2.41 |
| 32217 | c-JunL protein [Takifugu rubripes] | SIGENAE salmonids | 530 | 31.03 | 12.87 | 2.41 |
| 88973 | Salmo salar retinoic acid receptor gamma b (Rargb) gene, partial cds; and homeobox protein HoxC13ba (HoxC13ba), homeobox protein HoxC12ba (HoxC12ba), homeobox protein HoxC11ba (HoxC11ba), homeobox protein HoxC10ba (HoxC10ba), homeobox protein HoxC9ba (HoxC9ba), homeobox protein HoxC8ba (HoxC8ba), homeobox protein HoxC6ba (HoxC6ba), homeobox protein HoxC5ba (HoxC5ba), and homeobox protein HoxC4ba (HoxC4ba) genes, complete cds | SIGENAE salmonids | 569 | 15.33 | 6.52 | 2.35 |
| 87869 | PREDICTED: ankyrin repeat and SOCS box protein 2-like [X (Silurana) tropicalis] | Refseq metazoa | 1,003 | 47.34 | 20.40 | 2.32 |
| 30201 | importin subunit alpha-3 [D. rerio] | Refseq zebrafish | 798 | 32.26 | 13.94 | 2.31 |
| 89608 | troponin T3b, skeletal, fast isoform 1 [D. rerio] | SIGENAE salmonids | 615 | 884.84 | 385.33 | 2.30 |
| 35607 | Catechol O-methyltransferase [Salmo salar] | SIGENAE salmonids | 515 | 14.44 | 6.34 | 2.28 |
| 38552 | PREDICTED: nuclear pore complex protein Nup98-Nup96 [X (Silurana) tropicalis] | Refseq metazoa | 532 | 30.11 | 13.38 | 2.25 |
| 30783 | guanylate-binding protein [Oncorhynchus mykiss] | SIGENAE salmonids | 502 | 136.75 | 61.15 | 2.24 |
| 30106 | Oncorhynchus mykiss SYPG1 (SYPG1), PHF1 (PHF1), and RGL2 (RGL2) genes, complete cds; DNaseII pseudogene, complete sequence; LGN-like, PBX2 (PBX2), NOTCH-like, TAP1 (TAP1), and BRD2 (BRD2) genes, complete cds; and MHCII-alpha and Raftlin-like pseudogenes, complete sequence | SIGENAE salmonids | 673 | 39.31 | 17.85 | 2.20 |
| 31068 | Salmo salar homeobox protein HoxD13aa (HoxD13aa) gene, partial sequence; and homeobox protein HoxD13aa, homeobox protein HoxD12aa (HoxD12aa), homeobox protein HoxD11aa (HoxD11aa), homeobox protein HoxD10aa (HoxD10aa), homeobox protein HoxD9aa (HoxD9aa), homeobox protein HoxD4aa (HoxD4aa), homeobox protein HoxD3aa (HoxD3aa), and homeobox protein HoxD1aa (HoxD1aa) genes, complete cds | SIGENAE salmonids | 673 | 33.36 | 15.20 | 2.19 |
| 30351 | ---NA--- |  | 503 | 21.04 | 9.73 | 2.16 |
| 29191 | ---NA--- |  | 546 | 29.08 | 13.58 | 2.14 |
| 111991 | ---NA--- |  | 596 | 26.88 | 12.69 | 2.12 |
| 31654 | growth arrest-specific 7 [X laevis] | Refseq metazoa | 574 | 21.18 | 10.08 | 2.10 |
| 31714 | ---NA--- |  | 525 | 57.75 | 27.97 | 2.07 |
| 112409 | ---NA--- |  | 657 | 33.96 | 16.48 | 2.06 |
| 29336 | Mitoferrin-2 [Salmo salar] | SIGENAE salmonids | 848 | 38.79 | 18.89 | 2.05 |
| 88873 | protein arginine methyltransferase 7 [D. rerio] | SIGENAE salmonids | 580 | 13.07 | 6.39 | 2.04 |
| 67125 | PREDICTED: similar to KIAA1276 [Ornithorhynchus anatinus] | Refseq metazoa | 518 | 57.70 | 28.34 | 2.04 |
| 33682 | ---NA--- |  | 537 | 35.69 | 17.67 | 2.02 |
