# Supplementary material for: Deep RNA Sequencing of the Skeletal Muscle Transcriptome in Swimming Fish
Source: PLoS One. 2013 Jan 8;8(1):e53171. doi: 10.1371/journal.pone.0053171 (PMC3540090; doi:10.1371/journal.pone.0053171)
Supplement: Table S7 — Q-PCR validation of fold change (fc) expression by read-per-kilo-base-of-exon-model (RPKM) values of selected contigs in red and white muscle of swimmers. Contigs were selected based on whether they were larger than 500 nt, had a SIGENAE salmonid annotation and were differentially expressed at a fc ≤0.5 or fc ≥2. Columns represent contig annotation, sequence length of the contig, fold change (fc) expression of swimmers over resters by RPKM and by Q-PCR. Abbreviations: guanylate-binding protein (gbp); troponin T3b skeletal fast isoform 1 (T3b1); troponin C skeletal muscle (tropC); IgM membrane heavy bound form (IgM); retinoic acid receptor gamma b (Rargb); phosphofructokinase muscle b (pfkmb); follistatin-related protein 1 (fstl1); myoblast determination protein 2 (MyoD2); nuclear receptor coactivator 4 (ncoa4); growth hormone 2 (gh2); fatty acid binding protein 6 (fabp6); titin-like (ttn); four and a half LIM domains protein 1 (fhl1); ubiquitin specific protease 14 (ubp14); heat shock protein 30 (hsp30). (DOCX) [file pone.0053171.s011.docx]

**Table S7**.

| ***Red muscle*** |  |  |  |  |
| --- | --- | --- | --- | --- |
| **Contig number** | **Contig annotation** | **Sequence length** | **RPKM (fc)** | **Q-PCR (fc)** |
| 142086 | *gbp* | 533 | 9.62 | 4.87 |
| 114142 | *tropT3b1* | 762 | 0.41 | 1.30 |
| 36750 | *tropC* | 932 | 0.46 | 8.92 |
| 37437 | *IgM* | 744 | 4.99 | 2.49 |
| 38951 | *Rargb* | 928 | 0.47 | 1.31 |
| 40308 | *pfkmb* | 591 | 2.16 | 6.95 |
| 3031 | *fstl1* | 806 | 0.47 | 0.77 |
| 39111 | *Myod2* | 598 | 0.32 | 2.36 |
| 40144 | *ncoa4* | 643 | 2.05 | 4.75 |
| 40962 | *gh2* | 579 | 2.37 | 1.29 |
| 45473 | *fabp6* | 527 | 0.28 | 0.78 |
|  |  |  |  |  |
| ***White muscle*** |  |  |  |  |
| **Contig number** | **Contig annotation** | **Sequence length** | **RPKM (fc)** | **Q-PCR (fc)** |
| 30783 | *gbp* | 502 | 2.24 | 22.32 |
| 89608 | *tropT3b1* | 615 | 2.30 | 2.13 |
| 87861 | *tropC* | 605 | 2.60 | 6.59 |
| 28989 | *IgM* | 652 | 4.99 | 0.20 |
| 88973 | *Rargb* | 569 | 2.35 | 0.61 |
| 111827 | *pfkmb* | 1985 | 0.32 | 0.27 |
| 29086 | *ttn* | 2886 | 0.49 | 0.86 |
| 30004 | *fhl1* | 568 | 2.72 | 11.00 |
| 30465 | *ubp14* | 739 | 0.48 | 0.65 |
| 64953 | *hsp30* | 820 | 0.03 | 1.26 |
