# Supplementary material for: Deep RNA Sequencing of the Skeletal Muscle Transcriptome in Swimming Fish
Source: PLoS One. 2013 Jan 8;8(1):e53171. doi: 10.1371/journal.pone.0053171 (PMC3540090; doi:10.1371/journal.pone.0053171)
Supplement: Table S8 — Expression of reproductive-related contigs in red and white skeletal muscle of resting and swimming trout. Columns represent contig number, contig annotation, sequence length of the contig, read-per-kilo-base-of-exon-model (RPKM) value for swimmers, RPKM value for resters, fold change (fc) expression by RPKM. Abbreviations: estrogen receptor beta (erb); follicle-stimulating hormone beta (fshb); vitellogenin (vtg); kiss-1 receptor (kiss-1r). (DOCX) [file pone.0053171.s012.docx]

**Table S8**.

| ***Red muscle*** |  |  |  |  |  |
| --- | --- | --- | --- | --- | --- |
| **Contig**  **number** | **Contig annotation** | **Sequence length** | **RPKM swim** | **RPKM rest** | **RPKM (fc)** |
| *62717* | *erb* | 211 | 6.20 | 5.27 | 1.18 |
| *148410* | *fshb* | 213 | 9.83 | 13.91 | 0.71 |
| *48819* | *fshb* | 219 | 10.16 | 13.53 | 0.75 |
| *48091* | *fshb* | 228 | 5.16 | 5.96 | 0.87 |
| *141676* | *fshb* | 710 | 29.30 | 30.79 | 0.95 |
| *61785* | *fshb* | 211 | 14.26 | 11.71 | 1.22 |
| *808* | *fshb* | 200 | 17.01 | 8.64 | 1.97 |
|  |  |  |  |  |  |
| ***White muscle*** |  |  |  |  |  |
| **Contig**  **number** | **Contig**  **annotation** | **Sequence length** | **RPKM swim** | **RPKM rest** | **RPKM (fc)** |
| *28645* | *vtg* | 107 | 0 | 9.70 | 0 |
| *74667* | *vtg* | 114 | 0 | 9.11 | 0 |
| *111533* | *vtg* | 118 | 0 | 7.54 | 0 |
| *54553* | *vtg* | 243 | 0.58 | 19.53 | 0.03 |
| *60283* | *vtg* | 194 | 0.73 | 18.35 | 0.04 |
| *29589* | *vtg* | 742 | 4.62 | 91.54 | 0.05 |
| 96961 | *erb* | 136 | 3.15 | 6.54 | 0.48 |
| 64300 | *erb* | 124 | 2.31 | 4.78 | 0.48 |
| 27149 | *erb* | 107 | 4.01 | 5.54 | 0.72 |
| 11525 | *erb* | 105 | 6.81 | 7.06 | 0.96 |
| *52574* | *kiss1r* | 128 | 3.35 | 6.95 | 0.48 |
| 88566 | *fshb* | 680 | 23.13 | 18.97 | 1.22 |
| 32964 | *fshb* | 506 | 20.07 | 16.41 | 1.22 |
| 84557 | *fshb* | 141 | 5.07 | 1.05 | 4.82 |
